# Type I Interferons Function as Autocrine and Paracrine Factors to Induce Autotaxin in Response to TLR Activation

**DOI:** 10.1371/journal.pone.0136629

**Published:** 2015-08-27

**Authors:** Jianwen Song, Ming Guan, Zhenwen Zhao, Junjie Zhang

**Affiliations:** 1 Key Laboratory of Cell Proliferation and Regulation Biology, Ministry of Education, Institute of Cell Biology, College of Life Sciences, Beijing Normal University, Beijing, China; 2 Key Laboratory of Analytical Chemistry for Living Biosystems, Institute of Chemistry Chinese Academy of Sciences, Beijing, China; University of Hong Kong, HONG KONG

## Abstract

Lysophosphatidic acid (LPA) is an important phospholipid mediator in inflammation and immunity. However, the mechanism of LPA regulation during inflammatory response is largely unknown. Autotaxin (ATX) is the key enzyme to produce extracellular LPA from lysophosphatidylcholine (LPC). In this study, we found that ATX was induced in monocytic THP-1 cells by TLR4 ligand lipopolysaccharide (LPS), TLR9 ligand CpG oligonucleotide, and TLR3 ligand poly(I:C), respectively. The ATX induction by TLR ligand was abolished by the neutralizing antibody against IFN-β or the knockdown of IFNAR1, indicating that type I IFN autocrine loop is responsible for the ATX induction upon TLR activation. Both IFN-β and IFN-α were able to induce ATX expression via the JAK-STAT and PI3K-AKT pathways but with different time-dependent manners. The ATX induction by IFN-β was dramatically enhanced by IFN-γ, which had no significant effect on ATX expression alone, suggesting a synergy effect between type I and type II IFNs in ATX induction. Extracellular LPA levels were significantly increased when THP-1 cells were treated with IFN-α/β or TLR ligands. In addition, the type I IFN-mediated ATX induction was identified in human monocyte-derived dendritic cells (moDCs) stimulated with LPS or poly(I:C), and IFN-α/β could induce ATX expression in human peripheral blood mononuclear cells (PBMCs) and monocytes isolated form blood samples. These results suggest that, in response to TLR activation, ATX is induced through a type I INF autocrine-paracrine loop to enhance LPA generation.

## Introduction

Autotaxin (ATX), also known as ENPP2 (ectonucleotide pyrophosphatase phosphodiesterase-2), is a secreted glycoprotein with lysophospholipase D (lysoPLD) activity converting lysophosphatidylcholine (LPC) into lysophosphatidic acid (LPA)[1]. LPA is a bioactive phospholipid acting on specific G protein-coupled receptors to regulate a wide range of cellular activities, ranging from cell proliferation, differentiation, migration, to anti-apoptosis[2]. ATX functions as the key enzyme for LPA production in plasma [3–5]. Many, if not all, biological functions of ATX appear to be mediated by LPA signaling.

Increased ATX expression has been detected in several cancers, and the effects of ATX-LPA axis in cancers are extensively studied [2, 6, 7]. Meanwhile, emerging data indicate that ATX-LPA axis plays an important role in immunity. As an important phospholipid mediator in inflammation and immunity, LPA modulates immune response by attracting and activating T-cells, B-cells and macrophages directly or indirectly by influencing their interactions with other cell types[8–13]. ATX is constitutively expressed in the high endothelial venules (HEVs) and facilitates T cell entry into lymph nodes by stimulating transendothelial migration (TEM) [14–16]. Recently, it has been found that ATX expression and activities are increased in several inflammatory diseases. For example, ATX is induced in mouse lung bronchial epithelial cells and alveolar macrophages during the bleomycin-induced pulmonary inflammation and fibrosis [17, 18], and upregulation of ATX expression is observed in synovial fibroblasts from rheumatoid arthritis (RA) patient as well as in the mouse model of arthritis [19, 20]. Increased ATX activity levels are detected in serum of patient with hepatitis C [21], and ATX expression is significantly elevated in hepatitis-related hepatocellular cancer (HCC) compared to HCC tissues developed from non-inflammatory background [22]. However, the mechanism of ATX upregulation in inflammatory states is largely elusive.

The multiple IFN-α members and IFN-β belong to the type I interferons (IFNs), which are the first family of cytokines discovered and named for their potent ability to “interfere” with viral replication [23]. Type I IFNs function through the type I IFN receptor (IFNAR) composed of two subunits, IFNAR1 and IFNAR2 [24]. JAKs, TYK2, and STATs are the major downstream signaling molecules of the IFN pathway [25–27]. The Toll-like receptors (TLRs) comprise of a cellular system in response to a broad range of infections by bacteria, fungi, protozoa and viruses [28, 29]. Several TLR ligands, such as lipopolysaccharide (LPS, ligand for TLR4) and nucleic acids (ligands for TLR3, TLR7, TLR8 and TLR9), induce potent induction of type I IFNs through the activation of interferon-regulatory factors (IRFs) [30, 31]. Type I IFNs implicate in the induction of a significant proportion of genes regulated by TLR signaling, and function as key components in infection and inflammatory reaction [32].

We have recently demonstrated that ATX is induced in LPS-stimulated THP-1 cells [33, 34]. To further understand the regulation of ATX-LPA axis in immune responses, in this study we investigated the mechanism of ATX regulation by different TLR ligands in several immune cell types, including human peripheral blood mononuclear cells (PBMCs), monocytes, and monocyte-derived dendritic cells (moDCs). It was found that ATX was induced via the type I IFN autocrine loop during TLR activation, and that type I IFNs also could function as paracrine factors to induce ATX in immune cells. Our findings described a previously unknown function of type I IFNs in ATX induction and revealed the mechanism of ATX-LPA axis regulation in response to TLR activation.

## Material and Methods

### Cells culture and treatments

THP-1 cells were cultured in RPMI-1640 medium supplemented with fetal bovine serum (10%), L-glutamine (2 mM), streptomycin (100 μg/ml) and penicillin (100 U/ml) at 37°C in a humidified atmosphere containing 5% CO2. For experiments to detect the secreted ATX, THP-1 cells were cultured in a conditioned serum-free medium with 250 μg/ml fatty-acid free BSA as described previously [33]. Human peripheral blood mononuclear cells (PBMCs) were obtained from leukapheresis specimens of normal donors after Ficoll-Paque density gradient separation. PBMCs were washed twice in Hank’s balanced salt solution and then resuspended in RPMI-1640 medium supplemented with fetal bovine serum (10%). Highly enriched monocytes (>80% CD14+) were purified by adherence as described [35]. For the generation of dendritic cells, monocytes were cultured for 7 days in RPMI and 10% FBS supplemented with 50 ng/ml GM-CSF and 50 ng/ml IL-4 [36]. This study has been approved by the ethics committee of College of Life Sciences, Beijing Normal University. The donors who were enrolled in this study have signed the informed consent.

### Cytokines, antibodies and inhibitors

LPS (from Escherichia coli serotype 055:B5) and poly(I:C) was purchased from Sigma-Aldrich. CpG oligonucleotides were purchased from InvivoGen (San Diego, CA). Recombinant human IFN-α, IFN-β, IFN-γ, GM-CSF, IL-4 and TNF-α were purchased from PeproTech. JAK inhibitor pyridone 6 (P6) was obtained from Sigma-Aldrich. PI3-kinase inhibitor LY294002, extracellular signal-regulated kinase (ERK) inhibitor PD98059 and p38 MAPK inhibitor SB202190 were obtained from Calbiochem (La Jolla, CA). The primary antibodies against STAT3, IFNAR1 and β-actin were purchased from Santa Cruz Biotechnology (Santa Cruz, CA). The primary antibodies against STAT1, AKT, IRF3 and IRF7 were purchased from Cell Signalling Technology (Beverly, MA). The neutralizing antibody against IFN-α and that against IFN-β were purchased from PeproTech. The primary antibody against ATX was produced by our lab as described previously [33].

### RT-PCR and quantitative real-time RT-PCR (qRT-PCR)

Total RNA was extracted from cells with Trizol (Invitrogen), and then digested with DNase I (Ambion) for 15 min at 37°C in order to remove DNA contamination. RNA (2 μg) from cells were reverse-transcribed using anchored oligo dT primers and the Reverse Transcription System (Promega). The cDNAs encoding indicated genes were amplified with specific primers. ATX: forward primer, 5’-GACTATGACTAGGATGGCTTAC-3’ and reverse primer, 5’-GATGATGCTGTAGTAGTGAGT-3’; GAPDH: forward primer, 5’-TTAGCACCCCTGTCCAAGG-3’ and reverse primer, 5’-CCTACTCCTTGGAGGCCATG-3’; MX2: forward primer, 5’-AACTGTTCAGAGCACGATT-3’ and reverse primer, 5’-TTCCAAGAAGTAGGCATTCA-3’; IFNAR1: forward primer, 5’-TGCCATGCCAGAAGATAGTG-3’ and reverse primer, 5’-TTAGGTGCTCAGGCTTCCAG-3’; TLR3 forward primer, 5’-CAACAACAACATAGCCAACA-3’ and reverse primer, 5’-ACCTTCTTCTCAACGGATG-3’; TLR4 forward primer, 5’-TGGATACGTTTCCTTATAAG-3’ and reverse primer, 5’-GAAATGGAGGCACCCCTTC-3’; TLR9 forward primer, 5’-CAACATCCACAGCCAAGT-3’ and reverse primer, 5’-CAGGTAATTGTCACGGAGA-3’; IFN-αs, forward primer, 5’-GATGGCCGTGCTGGTGCTCA-3’[37] and reverse primer, 5’-TGATTTCTGCTCTGACAACCTCCC-3’; IFN-β, forward primer, 5’-AAACTCATGAGCAGTCTGCA-3’ and reverse primer, 5’-AGGAGATCTTCAGTTTCGGAGG-3’. TNF-α, forward primer, 5’-TCCAGACTTCCTTGAGACA-3’; and reverse primer, 5’-GGCGATTACAGACACAACT-3’. The qRT-PCR was performed using the iQ SYBR Green Supermix (BioRad) with the iCycler iQ realtime RT-PCR detection system (Bio-Rad). Relative expression of each target gene was estimated by normalization with the expression of GAPDH. Each qRT-PCR experiment was repeated at least three times with 3 parallel samples.

### Immunoblotting assays

Cells were lysed in RIPA buffer for 30 min. After centrifugation, the supernatants were quantified by bicinchoninic acid assay (Micro BCA; Pierce Biotechnology, Rockford, IL). For experiments detecting the secreted ATX protein, the culture medium was concentrated (by approximately 30-fold) using Amicon Ultra 30,000 (Millipore). Protein quantification was conducted and equal amount protein was loaded for each sample. Protein samples were subjected to SDS-PAGE and analyzed as described previously [33]. Each Western blot analysis was repeated at least three times.

### RNA interference

All siRNAs were synthesized in Gene-Pharma (Shanghai, China), and the target sequences were: IFNAR1: 5’-CTGGGATGGATAATTGGAT-3’; IRF3: 5’-CCACTTTGGTGTTTCATAA-3’ IRF7: 5’-GCCTCTATGACGACATCGA3’; STAT1: 5’-GCTTCTTGGTCCTAACGCC-3’; STAT3: 5’-CCACTTTGGTGTTTCATAA-3’; JAK1: 5’- GACAUGAUAUUGAGAACGA-3’; TYK2: 5’- GCAUCCACAUUGCACAUAA -3’; non-specific-siRNA (siNC): 5’-UUCUCCGAACGUGUCACGU-3’. The siRNAs were transfected into cells with lipofectamine 2000 (Invitrogen), according to the protocol supplied by manufacturer. In each siRNA transfection experiment, the siNC was used as control.

### Lipid extraction and analysis

Lipids were extracted by Bligh and Dyer method[38] with modification. In brief, 0.8 ml of conditioned medium were collected and used for extraction of LPA. The conditioned medium were mixed with 0.5 mL of PBS (1X) and 3 mL of a 1:2 (v/v) mixture of CHCl_3_: MeOH with HCl (10 μl, 6N). 14:0 LPA (10 pmol in 10 μL MeOH) was added as internal standard. The samples were vortexed and incubated on ice for 10 min. CHCl_3_ (1 ml) and H_2_O (0.5 ml) were then added and the samples were revortexed. The phases were separated by centrifugation (1,750 g for 10 min at 10°C) and the bottom phase was recovered. After solvent evaporation, the dried lipids were resuspended using 100 μl of MeOH for mass spectrometry (MS) analysis. Electrospray ionization-mass spectrometry and tandem mass spectrometry analyses were done using a QTRap 4500 Mass Spectrometer (Applied Biosystems/MDS SCIEX, Forster City, CA) as described previously [39].

### Detection of lysoPLD activity

The conditioned serum-free medium from THP-1 cells with or without exposure to IFN-α (50 ng/ml), IFN-β (10 ng/ml) or LPS (0.1 μg/ml) for 24h, by CpG ODN (1 μM) or poly(I:C) (10 μg/ml) for 12h was concentrated (40-fold) using Amicon Ultra 30,000 (Millipore). The lysoPLD activity in the concentrated conditioned medium was analyzed using fluorogenic substrate FS-3 as described previously [33]. Briefly, the assays were performed by mixing 50 μl concentrated medium with 10 μM FS-3 at 37°C for 4 h. LysoPLD activity was measured by detecting the fluorescence increase with 494 and 520 nm as the excitation and emission wavelengths, respectively.

## Results

### ATX induction in LPS-stimulated THP-1 cells is mediated by autocrine IFN-β production

**Fig 1:**
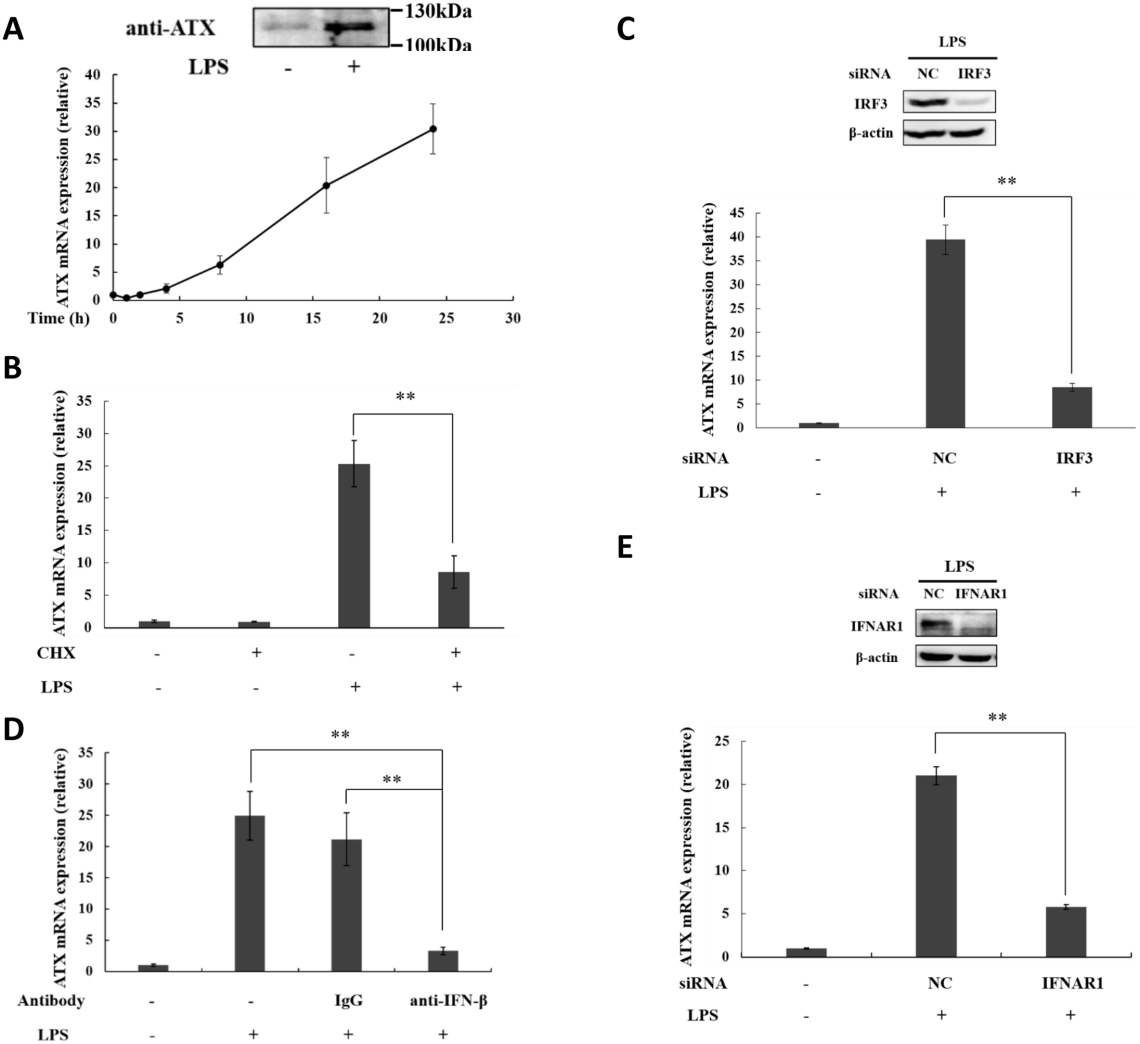
ATX is induced by TLR4 ligand LPS in THP-1 cells dependent on the IRF3-mediated autocrine IFN-β production. (A) THP-1 cells were stimulated with LPS (0.1 μg/ml) for the indicated times. ATX mRNA expression was detected by qRT-PCR. After LPS stimulation for 24h, the secreted ATX protein in culture medium was detected by Western blot. (B) THP-1 cells were incubated with CHX (1 μM) for 30 min prior to the stimulation with LPS. ATX mRNA expression was analyzed after LPS stimulation for 16h by qRT-PCR. (C) THP-1 cells were transfected with IRF3 siRNA and non-specific siRNA (siNC) respectively. After siRNA transfection for 48 h, THP-1 cells were treated with LPS for 16h. IRF3 protein was detected by Western blot, and ATX mRNA expression was analyzed by qRT-PCR. (D) THP-1 cells were preincubated for 30 min with IFN-β specific neutralizing antibody (anti-IFN-β; 1 μg/ml) or negative control antibody (rabbit IgG; 1 μg/ml), and then subjected to LPS stimulation. ATX mRNA expression was detected after LPS stimulation for 16h by qRT-PCR. (E) THP-1 cells were transfected with IFNAR1 siRNA and non-specific siRNA (siNC), respectively. After siRNA transfection for 48 h, THP-1 cells were treated with LPS for 16h. IFNAR1 protein was detected by Western blot, and ATX mRNA expression was analyzed by qRT-PCR. The ATX expression detected by qRT-PCR analyses was normalized to expression of GAPDH and presented relative to expression in untreated cells. All qRT-PCR data are expressed as mean values ± SD, n = 3. The p values derived from Student’s t test are (*) p < 0.05, (**) p < 0.01. A representative experiment out of three is shown.

In previous study, we have demonstrated that ATX is induced in human monocytic THP-1 cells under lipopolysaccharide (LPS) stimulation [33, 34]. LPS, as TLR4 ligand, induced a time- and dose-dependent ATX expression (Fig 1A, Figures A and B in S1 Fig). A significant induction of ATX was detected as early as 8h stimulation, and sustained to 24 h (Fig 1A). Cycloheximide (CHX), a translation inhibitor, significantly inhibited LPS-induced ATX expression (Fig 1B), suggesting that the ATX induction is dependent on new protein synthesis.

Type I interferons (IFNs) are produced in response to TLR activation and control the induction of significant proportion of genes regulated by TLR signaling [32]. IFN-β, but not IFN-α, was induced in the LPS-stimulated THP-1 cells (Figures A and B in S1 Fig). IFN-β is induced by LPS through the activation of IFN regulatory factors 3 (IRF3), which is phosphorylated and then translocated to the nucleus after TLR4 activation [40]. Knockdown of IRF3 by siRNA inhibited the ATX induction by LPS in THP-1 cells (Fig 1C). Furthermore, the neutralizing antibody against IFN-β significantly blocked ATX induction in LPS-stimulated THP-1 cells (Fig 1D). Type I IFNs, IFN-α and IFN-β, share a heterodimeric receptor IFNAR composed of IFNAR1 and IFNAR2 subunits. ATX induction by LPS was suppressed by the knockdown of IFNAR1 with siRNA (Fig 1E). These data indicate that the autocrine IFN-β production and the IFNAR signaling are required for the ATX induction in THP-1 cells in response to LPS stimulation.

### CpG oligonucleotides induce ATX expression through the IFN-β autocrine loop

**Fig 2:**
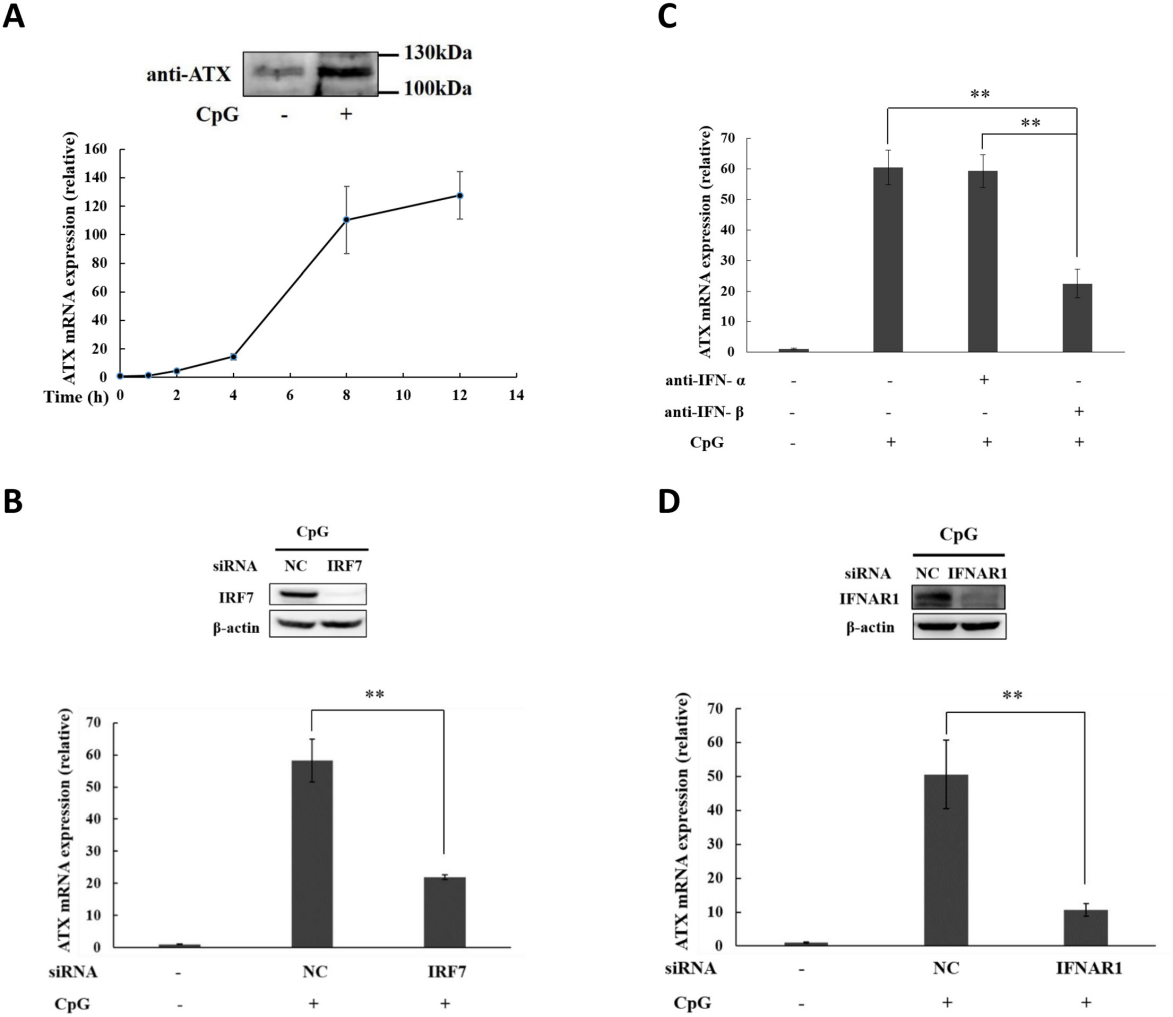
ATX is induced by TLR 9 ligand CpG oligonucleotides (ODN) in THP-1 cells dependent on the IRF7-mediated autocrine IFN-β production. (A) THP-1 cells were treated with CpG ODN (1 μM) for the indicated times. ATX mRNA expression was detected by qRT-PCR. After CpG ODN treatment for 12h, the secreted ATX protein in culture medium was detected by Western blot. (B) THP-1 cells were transfected with IRF7 siRNA and non-specific siRNA (siNC) respectively. After siRNA transfection for 48 h, THP-1 cells were treated with or without CpG ODN for 6h. IRF7 protein was detected by western blot, and ATX mRNA expression was analyzed qRT-PCR. (C) THP-1 cells were preincubated for 30 min with IFN-α specific neutralizing antibody (anti-IFN-α; 1 μg/ml) or IFN-β specific neutralizing antibody (anti-IFN-β; 1 μg/ml), and then subjected to CpG ODN treatment. ATX mRNA expression was detected after CpG ODN treatment for 6h by qRT-PCR. (D) THP-1 cells were transfected with IFNAR1 siRNA and non-specific siRNA (siNC), respectively. After siRNA transfection for 48 h, THP-1 cells were treated with CpG ODN for 6h. IFNAR1 protein was detected by Western blot, and ATX mRNA expression was analyzed by qRT-PCR. The ATX expression detected by qRT-PCR analyses was normalized to expression of GAPDH and presented relative to expression in untreated cells. All qRT-PCR data are expressed as mean values ± SD, n = 3. The p values derived from Student’s t test are (*) p < 0.05, (**) p < 0.01. A representative experiment out of three is shown.

The innate immune system of vertebrates is able to recognize unmethylated CG dinucleotides within the CpG motifs in microbial DNA via TLR9. Consequently, CpG oligonucleotides (ODN) can active the immune cells with TLR9 expression and induce cytokines to modulate immune responses [41]. Here, we found that ATX was induced in the CpG ODN-treated THP-1 cells in a time- and dose-dependent manner (Fig 2A, Figures C and D in S1 Fig). Type I IFNs, both IFN -α and IFN-β, were induced in the CpG ODN-treated THP-1 cells (Figures C and D in S1 Fig). CpG ODN induces type I IFN through the TLR9-IRF7 pathway [42]. Knockdown of IRF7 to block type I IFN production suppressed the ATX induction by CpG ODN (Fig 2B). The ATX induction in THP-1 cells in response to CpG ODN treatment was significantly inhibited by the neutralizing antibody against IFN-β, but not by the neutralizing antibody against IFN-α (Fig 2C). Moreover, the ATX induction by CpG ODN was inhibited by IFNAR1 knockdown (Fig 2D). These data indicate that ATX is induced through the IFN-β autocrine loop in CpG ODN-treated THP-1 cells.

### Poly(I:C) induces ATX expression through IRF3-mediated type I IFN production

**Fig 3:**
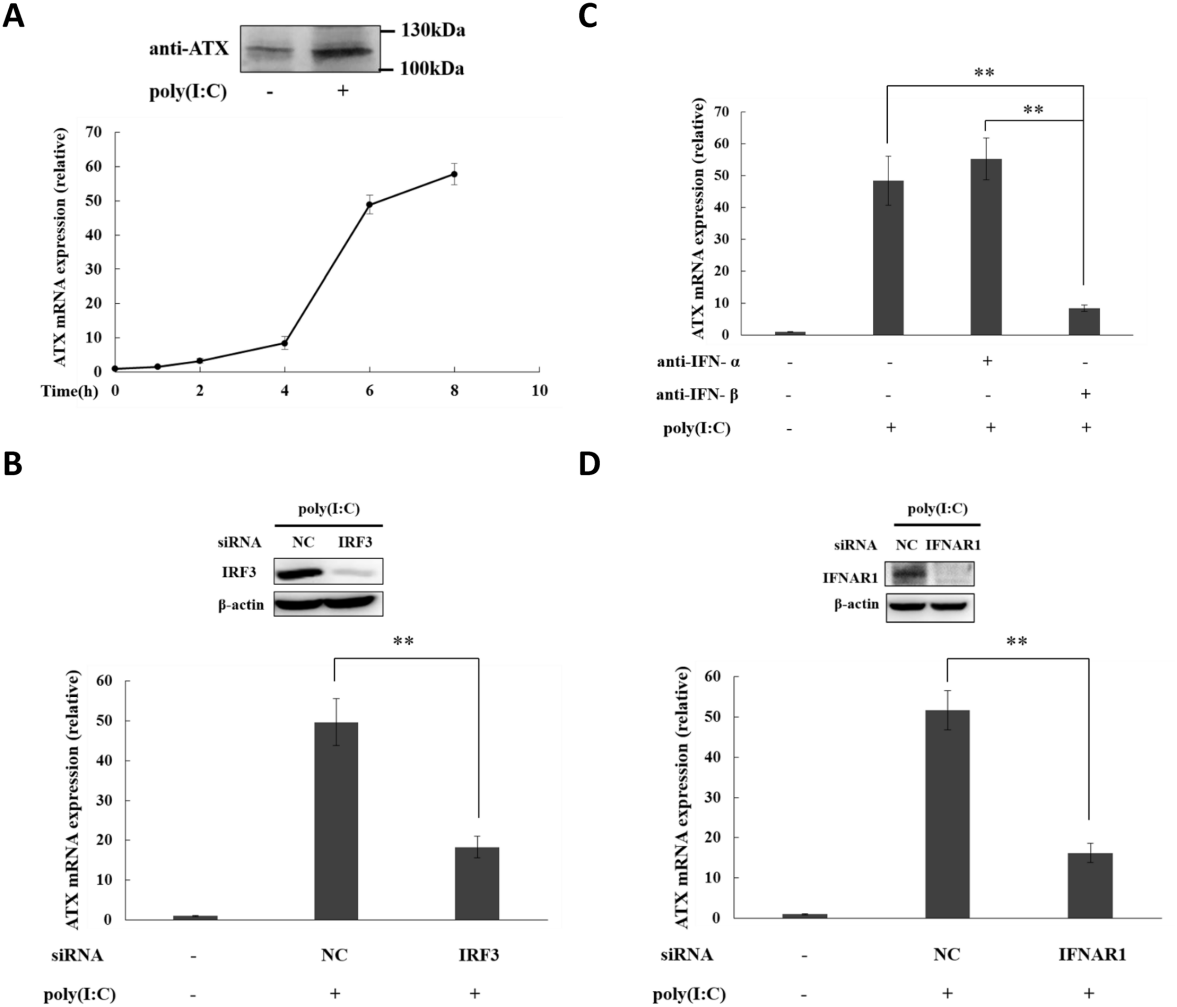
ATX is induced by TLR 3 ligand poly(I:C) in THP-1 cells dependent on the IRF3-mediated autocrine IFN-β production. (A) THP-1 cells were treated with poly(I:C) (10 μg/ml) for the indicated times. ATX mRNA expression was detected by qRT-PCR. After poly(I:C) treatment for 12 h, the secreted ATX protein in culture medium was detected by Western blot. (B) THP-1 cells were transfected with IRF3 siRNA and non-specific siRNA (siNC) respectively. After siRNA transfection for 48 h, THP-1 cells were treated with or without poly(I:C) for 6 h. IRF3 protein was detected by Western blot, and ATX mRNA expression was analyzed qRT-PCR. (C) THP-1 cells were preincubated with IFN-α specific neutralizing antibody (anti-IFN-α; 1 μg/ml) or IFN-β specific neutralizing antibody (anti-IFN-β; 1 μg/ml) for 30 min, and then subjected to poly(I:C) treatment. ATX mRNA expression was detected after poly(I:C) treatment for 6 h by qRT-PCR. (D) THP-1 cells were transfected with IFNAR1 siRNA and non-specific siRNA (siNC), respectively. After siRNA transfection for 48 h, THP-1 cells were treated with poly(I:C) for 6 h. IFNAR1 protein was detected by Western blot, and ATX mRNA expression was analyzed by qRT-PCR. The ATX expression detected by qRT-PCR analyses was normalized to expression of GAPDH and presented relative to expression in untreated cells. All qRT-PCR data are expressed as mean values ± SD, n = 3. The p values derived from Student’s t test are (*) p < 0.05, (**) p < 0.01. A representative experiment out of three is shown.

TLR3 recognizes viral double-stranded RNA (dsRNA) during infection. Polyriboinosinic:polyribocytidylic acid (poly(I:C)) is the synthetic analog of dsRNA, and functions as TLR3 ligand to active immune cells [43]. ATX induction was observed in the poly(I:C)-treated THP-1 cells (Fig 3A, Figures E and F in S1 Fig), and suppressed by IRF3 knockdown (Fig 3B), which blocks the type I IFN production by poly(I:C) via TLR3 activation [44]. Both IFN-β and IFN-α were induced in poly(I:C)-treated THP-1 cells (Figures E and F in S1 Fig). The neutralizing antibody against IFN-β significantly inhibited the ATX induction in poly(I:C)-treated THP-1 cells, but the neutralizing antibody against IFN-α did not (Fig 3C). Knockdown of IFNAR1 blocked the ATX induction by poly(I:C), suggesting the IFNAR signaling is essential for ATX induction (Fig 3D). These data indicate that the TLR3 ligand poly(I:C) induces ATX in THP-1 cells through the IRF3-mediated IFN-β production and the IFNAR signaling.

### Type I IFNs induce ATX expression through the JAK-STAT and PI3K-AKT signaling pathways

**Fig 4:**
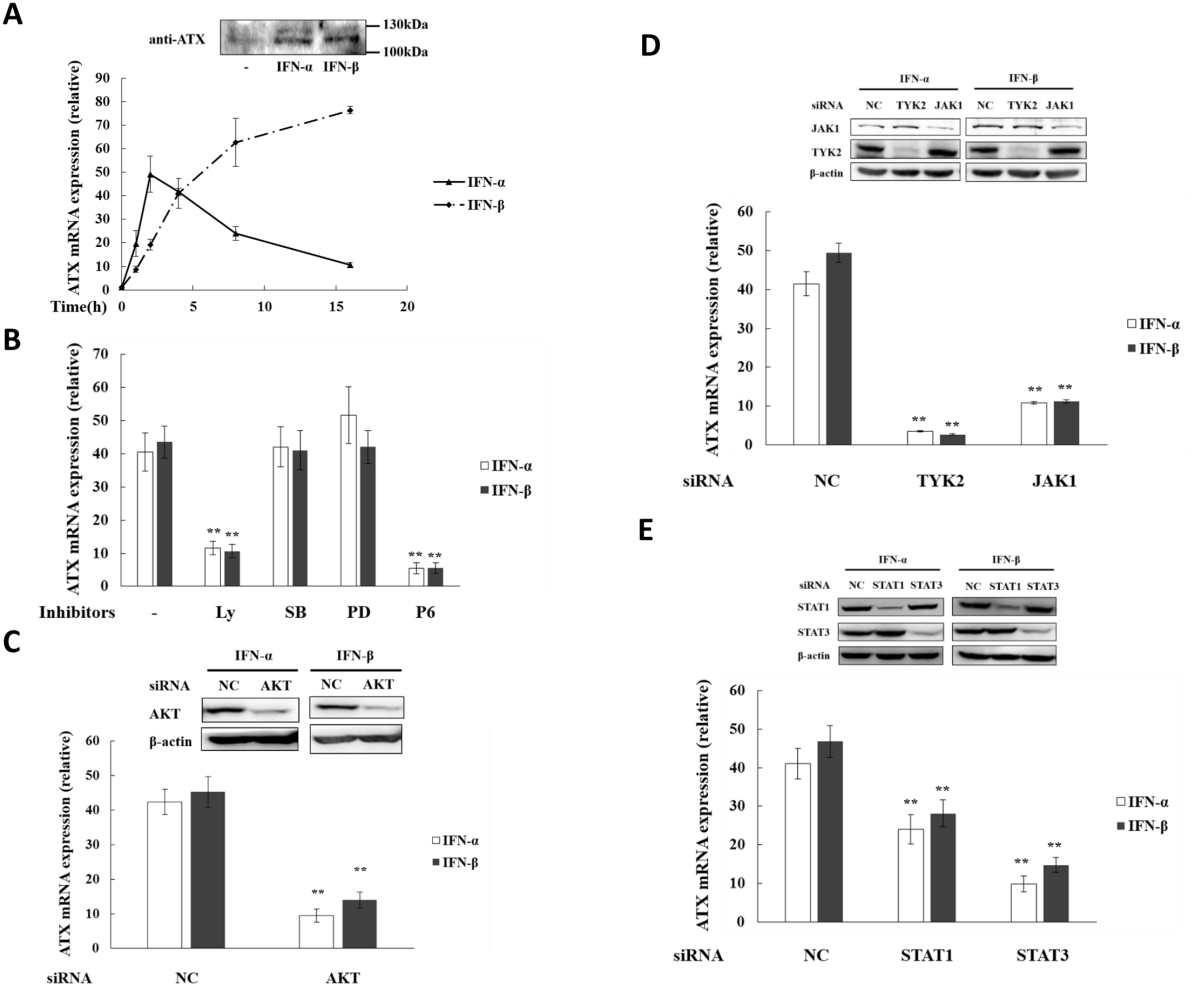
Type I interferons induce ATX expression in THP-1 cells through JAK-STAT and PI3K-AKT pathway. (A) THP-1 cells were treated with IFN-α (50 ng/ml) or IFN-β (10 ng/ml) for the indicated times. After treatment with IFN-α for 4 h or IFN-β for 6 h, the secreted ATX protein in culture medium was detected by Western blot. (B) THP-1 cells were treated with LY294002 (10 μM), SB202190 (10 μM), PD98059 (20 μM), pyridone 6 (P6; 10 μM) for 30 min before IFN-α or IFN-β treatment. After IFN-α treatment for 2 h or IFN-β treatment for 4 h, ATX mRNA levels were detected by qRT-PCR. (C) THP-1 cells were transfected with AKT siRNA and non-specific siRNA (siNC) respectively. After siRNA transfection for 48 h, THP-1 cells were treated with IFN-α for 2 h or with IFN-β for 4 h. AKT protein was detected by Western blot, and ATX mRNA expression was analyzed by qRT-PCR. (D) TYK2 and JAK1 siRNAs were transfected into THP-1 cells respectively, with non-specific siRNA (siNC) as the control. After siRNA transfection for 48 h, THP-1 cells were treated with IFN-α for 2 h or with IFN-β for 4 h. TYK2 and JAK1 were detected by Western blot, and ATX mRNA expression was analyzed by qRT-PCR. (E) STAT1 and STAT3 siRNAs were transfected into THP-1 cells respectively, with non-specific siRNA (siNC) as the control. After siRNA transfection for 48 h, THP-1 cells were treated with IFN-α for 2 h or with IFN-β for 4 h. STAT1 and STAT3 were detected by Western blot, and ATX mRNA expression was analyzed by qRT-PCR. The ATX expression detected by qRT-PCR analyses was normalized to expression of GAPDH and presented relative to expression in untreated cells. All qRT-PCR data are expressed as mean values ± SD, n = 3. The p values derived from Student’s t test are (*) p < 0.05, (**) p < 0.01. A representative experiment out of three is shown.

In order to detect whether type I IFN (IFN-α/β) can induce ATX expression directly, the THP-1 cells were treated with IFN-α and IFN-β respectively. It was found that both IFN-α and IFN-β induced ATX expression in THP-1 cells but with different time-dependent manners (Fig 4A). With IFN-α treatment, ATX expression was induced quickly, peaked at 2h of treatment and then decreased. However, with IFN-β treatment, ATX expression was increased continually, achieved high levels after 8h treatment and then kept at the high levels. The reason for such a difference between the ATX induction by IFN-α and IFN-β remains to be further clarified.

The binding of IFN-α/β to IFNAR induces activation of the receptor-associated Janus protein tyrosine kinases (TYK2 on IFNAR1 and JAK1 on IFNAR2), leading to the phosphorylation of STATs, which are involved in type I IFN-mediated induction of gene expression [27]. IFN-α/β-induced ATX expression in THP-1 cells was inhibited when Jak signaling was blocked by its specific inhibitor pyridone 6 (P6) or by Jak1/Tyk2 knockdown (Fig 4B and 4D). The ATX induction by IFN-α/β was dramatically inhibited by STAT3 knockdown, and partially inhibited by STAT1 knockdown (Fig 4E). PI3K is regarded as an upstream molecule implicated in IFN-mediated signaling. When PI3K-AKT signaling pathway was blocked in the presence of LY294002, the specific kinase inhibitor for PI3K, or by the knockdown of AKT, IFN-α/β -induced ATX expression in THP-1 cells was also inhibited (Fig 4B and 4C). However, PD98059 (an inhibitor for ERK activation) and SB202190 (an inhibitor for p38-mediated signaling) could not inhibit the IFN-α/β-induced ATX expression in THP-1 cells (Fig 4C). These results suggest that the activation of JAK-STAT and PI3K-AKT pathways, but not p38 MAPK and ERK signaling, is required for the ATX induction by IFN-α/β in THP-1 cells. Accordingly, the activation of JAK-STAT and PI3K-AKT pathways was also essential for the ATX induction in LPS-stimulated THP-1 cells (S2 Fig).

### The synergistic effects of IFN-γ on ATX induction by IFN-β

**Fig 5:**
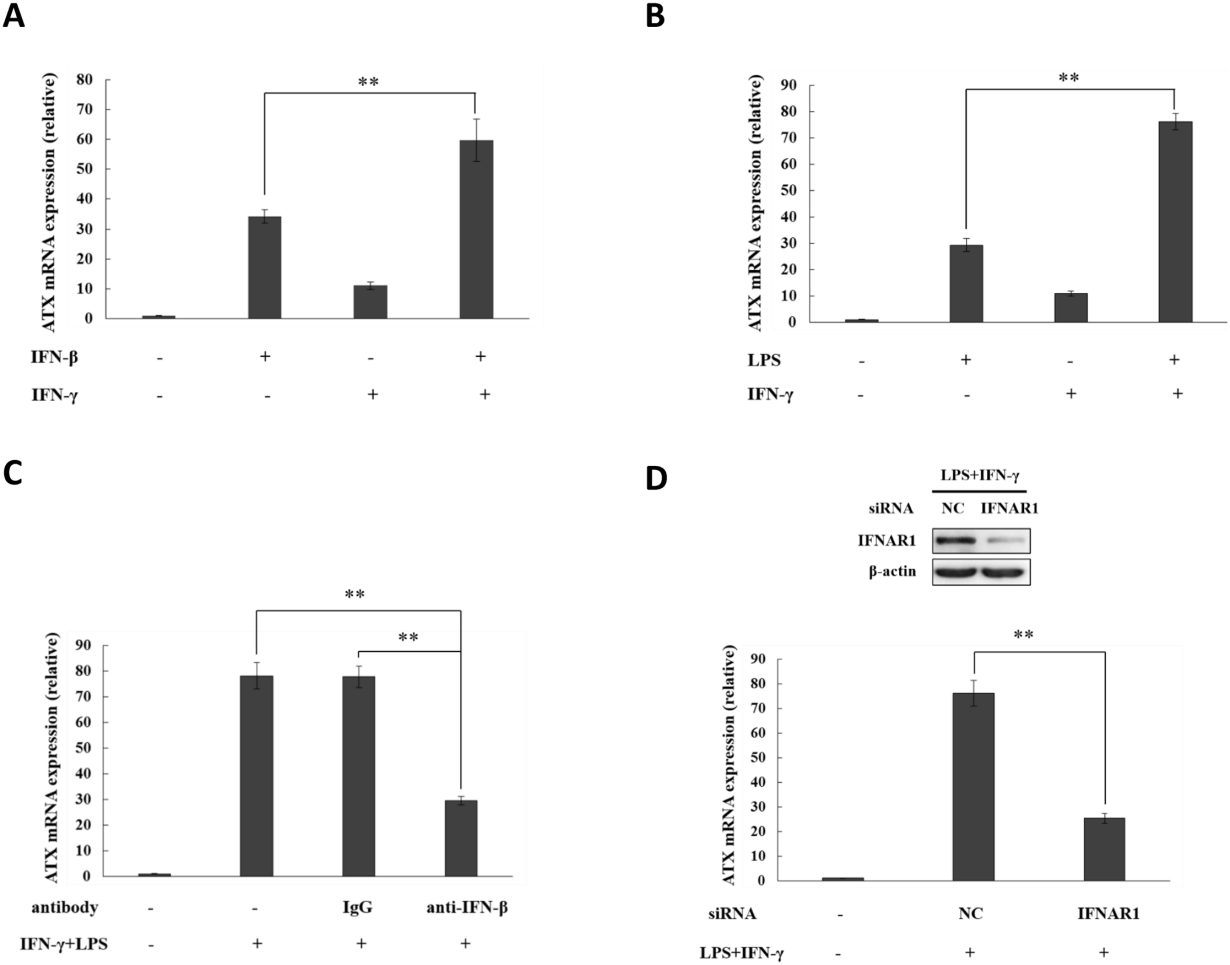
The synergistic effect of IFN-γ on the ATX induction by IFN-β. (A) THP-1 cells were treated for 4h with IFN-β (10 ng/ml) and/or IFN-γ (50 ng/ml) as indicated. ATX mRNA levels were detected by qRT-PCR. (B) THP-1 cells were stimulated by LPS for 16 h with or without IFN-γ priming. ATX mRNA levels were detected by qRT-PCR. (C) THP-1 cells were preincubated with IFN-β specific neutralizing antibody (anti-IFN-β; 1μg/ml) or negative control antibody (rabbit IgG; 1 μg/ml) for 30 min, and then subjected to LPS and/or IFN-γ treatment as indicated. ATX mRNA levels were detected by qRT-PCR after treatment for 16 h. (D) IFNAR1 siRNA and non-specific siRNA (siNC) were transfected into THP-1 cells respectively. After siRNA transfection for 48 h, THP-1 cells were treated with LPS plus IFN-γ for 16 h. IFNAR1 were detected by Western blot, and ATX mRNA expression was analyzed by qRT-PCR. The ATX expression detected by qRT-PCR analyses was normalized to expression of GAPDH and presented relative to expression in untreated cells. All qRT-PCR data are expressed as mean values ± SD, n = 3. The p values derived from Student’s t test are (*) p < 0.05, (**) p < 0.01. A representative experiment out of three is shown.

Type-I IFN (IFN-α/β) is produced in response to TLR activation at the early stages of the innate immune response. It is important to recognize whether IFN-α/β has the synergistic or antagonistic interaction with other cytokines in ATX expression regulation. The crosstalk between type I and type II IFN (IFN-γ) signaling has been reported previously [45, 46]. Here we found that, compared with IFN-β, IFN-γ itself has a very week ability to induce ATX in THP-1 cells, but the ATX induction by IFN-β was significantly enhanced in the presence of IFN-γ (Fig 5A). When the THP-1 monocytic cells were primed by IFN-γ, the ATX induction by LPS was also dramatically promoted (Fig 5B). The enhanced ATX induction was suppressed by the neutralizing antibody against IFN-β and knockdown of IFNAR1 (Fig 5C and 5D). These data suggest that IFN-γ has synergistic effects on the ATX induction by IFN-β.

### LPA production is enhanced during TLR activation and IFN-α/β treatment

**Fig 6:**
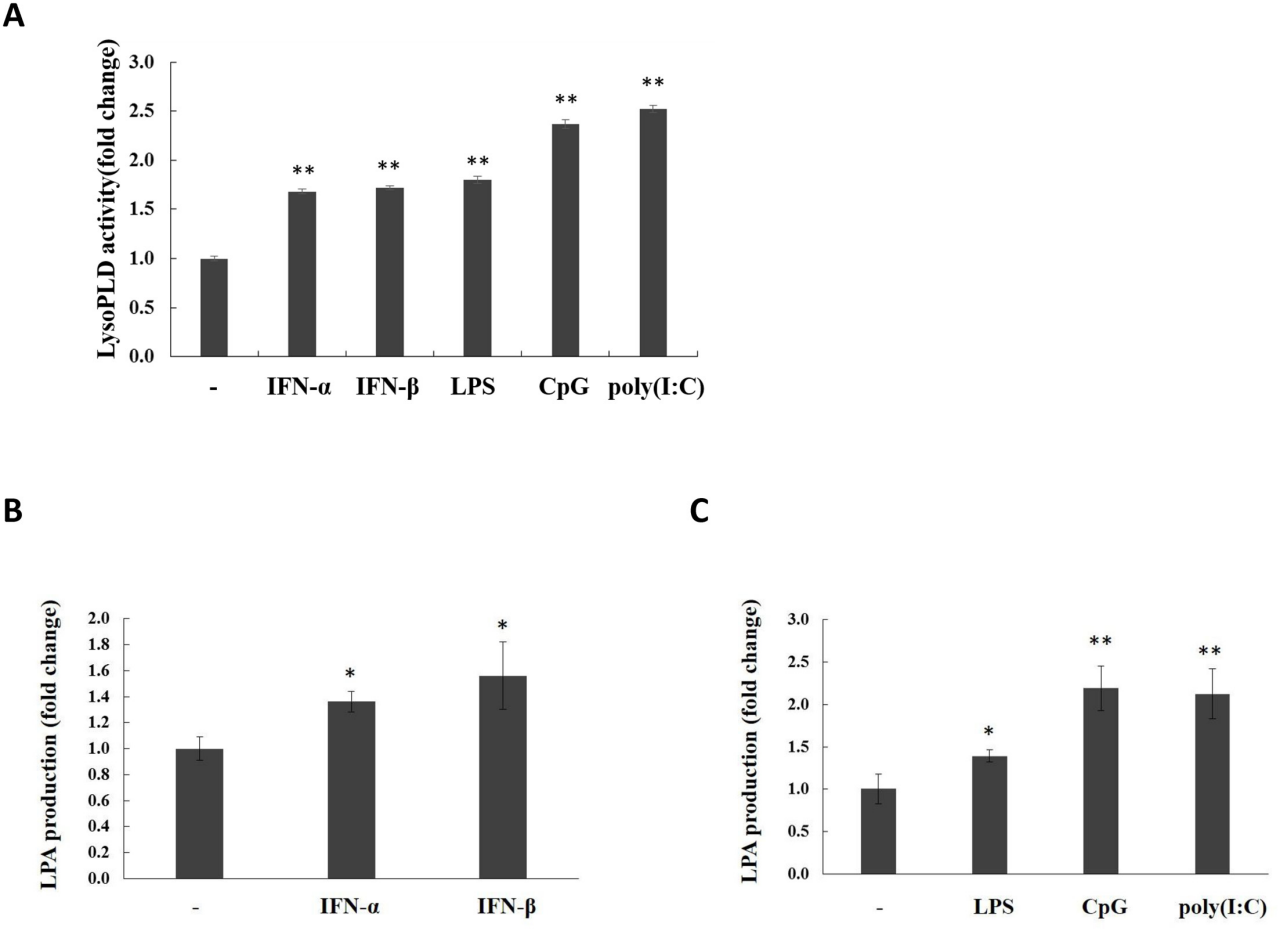
LysoPLD activity and LPA production were enhanced by IFN-α/β treatment and TLR activation. THP-1 cells were washed by PBS for three times and cultured with serum-free RPMI 1640, then stimulated by IFN-α (50 ng/ml), IFN-β (10 ng/ml) or LPS (0.1 μg/ml) for 24 h, by CpG ODN (1 μM) or poly(I:C) (10 μg/ml) for 12 h. (A) After stimulation, the lysoPLD activity in concentrated (40-fold) conditioned serum-free culture medium of THP-1 cells was analyzed using FS-3 as substrate. The conditioned serum-free medium of unstimulated THP-1 cells was concentrated (40-fold) and used as controls. (B, C) LPA levels in the supernatant of conditional medium were assayed by mass spectrometry. Data represent the mean and SD of triplicate determinations. The p values derived from Student’s t test are (*) p < 0.05, (**) p < 0.01.

ATX is a secreted lysoPLD hydrolyzing membrane-derived or albumin-bound LPC to produce extracellular LPA production [47]. With the ATX induction in THP-1 cells, the LysoPLD activity in cell culture was significantly increased during TLR activation or IFN-α/β treatment (Fig 6A). In order to investigate the effect of ATX upregulation on extracellular LPA levels, THP-1 cells cultured with serum-free medium were treated with IFN-α/β, LPS, CpG ODN, or poly(I:C) respectively. After stimulation, the concentration of major LPA species (16:0, 18:0, and 18:1 LPA) in THP-1 cell culture supernatants was detected by electrospray ionization mass spectrometry (ESI-MS) analyses. It was found that the ATX inducted by either IFN-α/β, LPS, CpG ODN or poly(I:C) treatment significantly increased the concentration of total LPA (Fig 6B and 6C) as well as that of 16:0, 18:0 and 18:1 LPA individually in cell culture medium with membrane-derived LPC as substrate (S1 Table). In addition, as the albumin-bound LPC is the available ATX substrate in blood, we tested the effects of TLR ligand stimulation and IFN-α/β treatment on LPA production in the presence of additional LPC and BSA. The increase of LPA levels in cell culture medium were also detected (S3 Fig). These results suggest that the extracellular LPA generation is enhanced during the TLR activation or IFN-α/β treatment.

### Type I IFNs function as autocrine factors to induce ATX in moDCs in response to TLR activation

**Fig 7:**
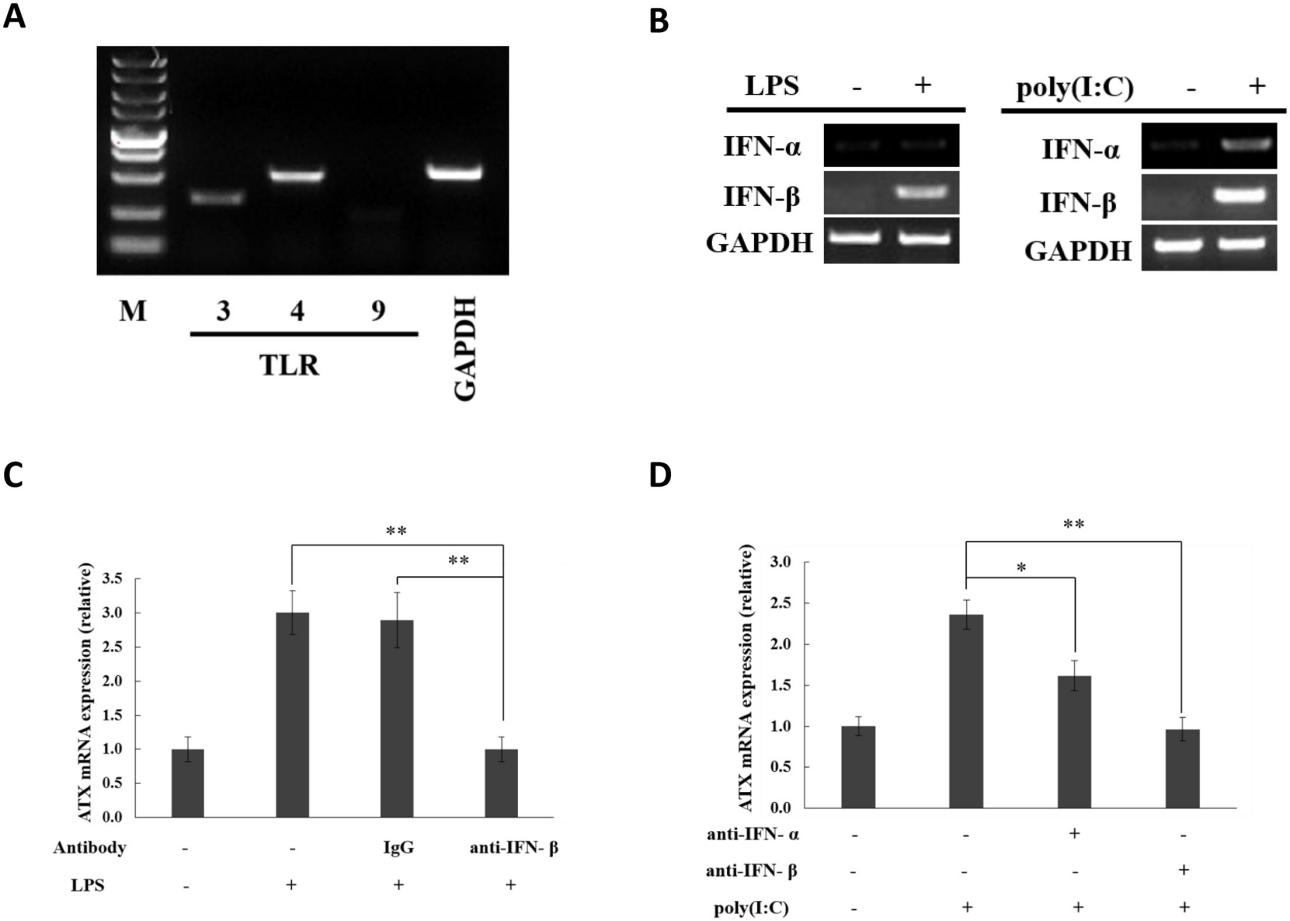
ATX is induced by LPS and poly(I:C) in moDCs through type I interferon autocrine loop. (A) TLR3, TLR4 and TLR9 mRNA expression in moDCs were analyzed by RT-PCR. (B) MoDCs were treated with LPS for 1 h or with poly(I:C) for 6h, and then IFN-α and IFN-β mRNA levels were detected by RT-PCR. (C) MoDCs were preincubated with IFN-β specific neutralizing antibody (anti-IFN-β; 1μg/ml) or negative control antibody (rabbit IgG; 1 μg/ml) for 30 min, and then subjected to LPS (0.1 μg/ml) stimulation. ATX mRNA expression was detected by qRT-PCR after LPS stimulation for 16 h. (D) THP-1 cells were preincubated with IFN-α specific neutralizing antibody (anti-IFN-α; 1μg/ml) or IFN-β specific neutralizing antibody (anti-IFN-β; 1μg/ml) for 30 min, and then subjected to poly(I:C) (10 μg/ml) treatment. ATX mRNA expression was detected by qRT-PCR after poly(I:C) treatment for 6 h. The ATX expression detected by qRT-PCR analyses was normalized to expression of GAPDH and presented relative to expression in untreated cells. All qRT-PCR data are expressed as mean values ± SD, n = 3. The p values derived from Student’s t test are (*) p < 0.05, (**) p < 0.01. A representative experiment out of three is shown.

Human peripheral blood mononuclear cells (PBMCs) were isolated from blood samples, and then monocytes were further separated from PBMCs. Monocyte-derived dendritic cells (moDCs) were obtained by culturing blood monocytes in the presence of GM-CSF and IL-4 as described previously [36]. The expression of TLR3 and TLR4, but not TLR9, was detected in moDCs by RT-PCR (Fig 7A). IFN-β was produced when human moDCs were stimulated with the TRL4 ligand LPS (Fig 7B). ATX induction was detected in the LPS-stimulated moDCs and suppressed by the neutralizing antibody against IFN-β (Fig 7C). Both IFN-α and IFN-β were produced when human moDCs were treated with the TRL3 ligand poly(I:C) (Fig 7B). The significant induction of ATX was also observed in the poly(I:C)-stimulated moDCs. The neutralizing antibody against IFN-β, as well as the neutralizing antibody against IFN-α, could suppressed the ATX induction by poly(I:C) in moDCs (Fig 7D). These data suggest that type I IFNs function as autocrine factors to induce ATX expression in moDCs in response to TLR activation.

### Type I IFNs induce ATX in human PBMCs and monocytes isolated from blood samples

**Fig 8:**
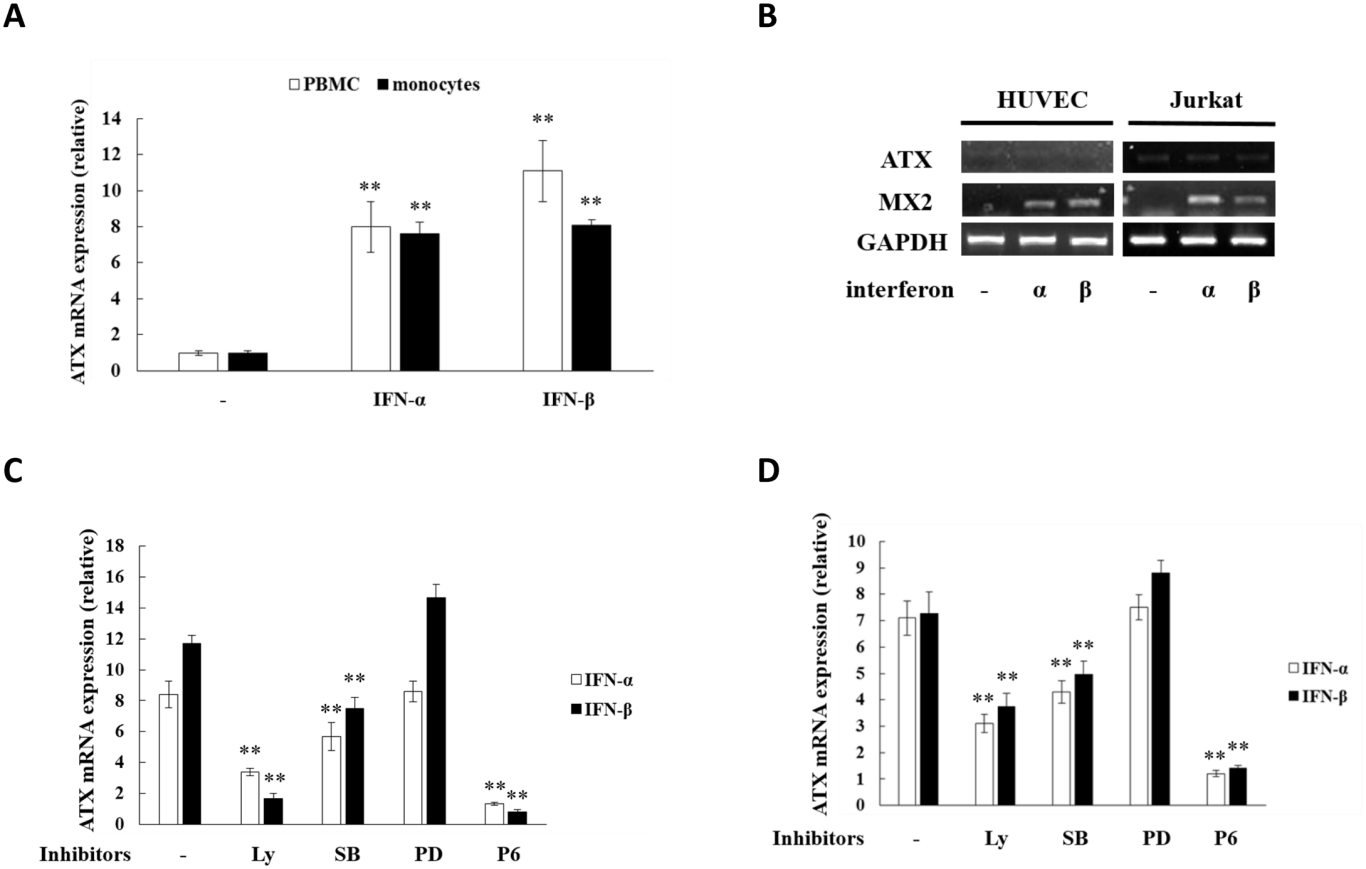
Type I interferons induce ATX expression in human PBMCs and monocytes. (A)Human PBMCs and monocytes were treated with IFN-α (50 ng/ml) for 2h or with IFN-β (10 ng/ml) for 4 h, and then ATX mRNA expression was detected by qRT-PCR. (B) HUVECs and Jurkat cells were treated with IFN-α (50 ng/ml) for 2 h or with IFN-β (10 ng/ml) for 4 h, and then ATX mRNA expression was detected by RT-PCR. (C, D)Human PBMCs and monocytes were pretreated with LY294002 (10 μM), SB202190 (10 μM), PD98059 (20 μM), or pyridone 6 (P6; 10 μM) for 30 min before the IFN-α/β treatment. After IFN-α treatment for 2 h or IFN-β treatment for 4 h, ATX mRNA levels were detected by qRT-PCR. The ATX expression detected by qRT-PCR analyses was normalized to expression of GAPDH and presented relative to expression in untreated cells. All qRT-PCR data are expressed as mean values ± SD, n = 3. The p values derived from Student’s t test are (*) p < 0.05, (**) p < 0.01. A representative experiment out of three is shown.

When type I IFN production is triggered by TLR activation, the type I IFNs (IFN-α and IFN-β) can function as autocrine and paracrine factors to regulate the gene expression in immune cells. In this study, human PBMCs and monocytes were isolated from blood samples and subjected to type I IFN treatment. ATX was expressed at low levels in human PBMCs and monocytes, but significantly induced by both IFN-α and IFN-β treatment (Fig 8A). Consistent with the results obtained above in IFN-α/β-treated THP-1 cells, ATX induction by IFN-α/β in PBMCs and monocytes was inhibited by the JAK inhibitor P6 and the PI3K inhibitor LY294002, indicating that activation of JAK-STAT and PI3K-AKT pathways is essential for the ATX induction. As to the MAPK inhibitors, ATX induction by IFN-α/β in PBMCs and monocytes was suppressed by SB202190, the inhibitor for p38-mediated signaling, but not by PD98059, an inhibitor for ERK activation (Fig 8C and 8D). Type I IFN receptor (IFNAR) is ubiquitously expressed. However, when HUVEC, Jurkat and several other cells were subjected to IFN-α/β treatment, MX2, the target gene of type I IFN, was induced, but ATX expression was not upregulated at all (Fig 8B, S4 Fig), suggesting that the ATX induction by IFN-α/β has cell type specificity.

## Discussion

LPA is a pleiotropic lipid molecule mediating various physiological and pathological activities through interaction with its receptors on cell membrane [48]. LPA receptors are expressed in most immune cells, including T-cells, B-cells, NK cells, mast cells, eosinophils, neutrophils, macrophages and monocytes [49]. As an important phospholipid mediator in immune responses, LPA is able to enhance the motility of human and mouse T cells [50], stimulate the production of reactive oxygen metabolites, integrin upregulation and chemotaxis in human eosinophils [51], and enhance maturation and cytokine production in immature dendritic cells (DCs) [52, 53]. However, the mechanism of LPA production regulation during immune responses remains unclear. ATX is a secreted glycoprotein with lysoPLD activity, functioning as the enzyme to produce most extracellular LPA from LPC [54]. Here we demonstrated that ATX was induced in human monocytic THP-1 cells by TLR3 ligand poly(I:C), TLR4 ligand LPS and TLR9 ligand CpG ODN, respectively. ATX induction was also observed in human moDCs treated with poly(I:C) or LPS. TLR system is responsible the recognition of infectious and perhaps other agents to initiate the inflammatory progress [55]. The ATX induction in response to TLR activation would increase the lysoPLD activity and enhance LPA levels in microenvironment, to modulate the activity of immune cells through the activation of LPA-LPA receptor signaling. Three alternative splicing isoforms of ATX, named α, β and γ, have been reported. In our previous study, we had characterized that β isoform ATX is expressed and induced in LPS-simulated THP-1 cells [33]. ATX in the conditional culture medium of THP-1 cells stimulated by LPS, CpG or poly(I:C) was detected as one band with molecular weight at approximately 120 kDa by Western-blot analysis (S4 Fig). It is interesting to note that ATX mRNA level and lysoPLD activity in culture medium are correlated, but do not have a linear relationship, suggesting there is a regulation of ATX activity at the post-transcriptional level, which need to be further studied in the future.

Type I interferons (IFNs) are produced during TLR activation and control the induction of a significant proportion of genes regulated by TLR signaling [32]. In this study, we found that type I IFN autocrine loop played a key role for the ATX induction in response to TLR activation. The IRF-mediated IFN-β production was required for the ATX induction in THP-1 cells under LPS, poly(I:C) or CpG ODN stimulation. Type I interferons (IFN-α/β) function as autocrine factors to upregulate ATX expression in human moDCs treated by LPS or poly(I:C). It has been reported that type I IFNs can be induced by the activation of TLRs 4,3,7,8 and 9 in immune cells [32]. The dendritic cells (DCs), especially the plasmacytiod DCs (pDCs), are regarded as the major cells for the type I-IFN production in response to TLR activation [56, 57]. Both mouse and human pDCs express high levels of TLR7 and TLR9 [58]. Whether ATX is induced in pDCs by TLR ligand through INF-α/β autocrine loop will be studied in the future. In addition to TLR activation, type I IFN production can be induced by dsRNA through the cytosolic receptors MDA5 and RIG-1 or by the right-handed conformation dsDNA (B-DNA) through DAI [59]. ATX expression in immune cells may also be induced by these stimulations.

Type I IFNs not only function as autocrine factors, but also as paracrine factors to regulate the immune response. In this study, it was found that both IFN-α and IFN-β was able to induce ATX expression in THP-1 cells, as well as in human PBMCs and monocytes isolated from blood sample. The IFNAR-mediated activation of JAK-STAT and PI3K-AKT pathways was required for the ATX induction by IFN-α and IFN-β, suggesting that IFN-α and IFN-β have somehow similar signaling mechanisms to induce ATX expression. However, IFN-α and IFN-β induced ATX in THP-1 cells with different time-dependent manners (Fig 4A). ATX expression was induced quickly by IFN-α, peaked at 2h of treatment and then decreased, while during IFN-β treatment ATX expression was increased continually, achieved high levels after 8h treatment and then kept at the high levels. It has been reported that IFN-β has higher receptor binding affinity than IFN-α [60, 61]. Although the relative affinity to receptor subunits is proposed as the reason for the differential activities of type I IFNs, the mechanisms of the different ATX induction activities between IFN-α and IFN-β remain to be further elucidated. Type I IFN receptor (IFNAR) is ubiquitously expressed. In HUVEC, Jurkat, HEK293, SW480, A549, and MCF-7 cells, IFN-α/β induced the expression of its target gene MX2, but not the expression of ATX (Fig 7B, S5 Fig), indicating that the ATX induction by IFN-α/β has cell type specificity.

ATX is originally regarded as a protein involved in cancer cell motility [62]. Recently, emerging data indicate that ATX-LPA axis has an important role in immunity. The enhanced ATX-LPA signaling is implicated in several inflammatory diseases, such as rheumatoid arthritis [20], asthma [63], pulmonary fibrosis [17] and hepatitis C [21]. It has been reported that TNF-α increased ATX production from synovial fibroblasts in both mouse and human arthritic joints [19]. In this study, we found that TNF-α could not induce ATX in THP-1 cells, but ATX was significantly induced in THP-1 cells by the treatment with TNF-α and IFN-γ together. It is interesting that the ATX induction by TNF-α plus IFN-γ was suppressed by the IFN-β specific neutralizing antibody or the knockdown of IFNAR1, suggesting that type I IFN plays an essential role in this process (Figures A, B and C in S6 Fig). It has been reported that TNF-α can slightly induce the expression of IFN-β in bone marrow-derived macrophages (BMDMs) from C57BL/6 mice [64]. A slight induction of IFN-β by TNF-α was observed in THP-1 cells (Figure D in S6 Fig), and the ATX induction by TNF-α-induced IFN-β may be further enhanced in the presence of IFN-γ.

## Conclusions

**Fig 9:**
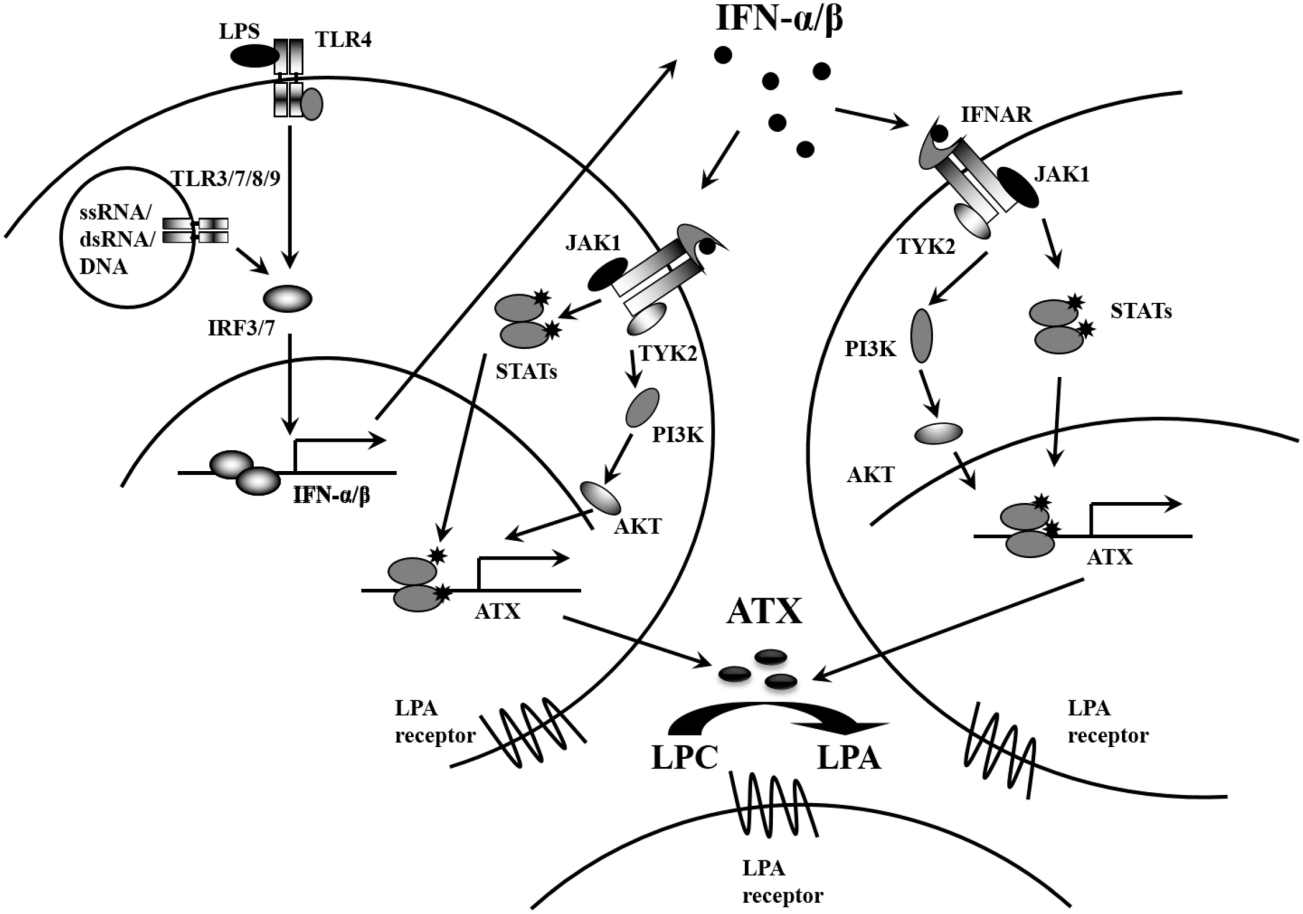
A proposed model of the ATX induction through type I IFN autocrine and paracrine loops in response to TLR activation. IFN-α/β was induced during TLR activation through IRF3/7, and then IFN-α/β functions as autocrine and paracrine factor to induced ATX expression in immune cells through the IFNAR-mediated JAK-STAT and PI3K-AKT pathways. The induction of ATX enhances LPA production in cellular microenvironment and activates the LPA-LPA receptor signaling to modulate immune responses.

In conclusion, we have demonstrated ATX is induced in response to TLR activation, and that type I IFN autocrine loop is essential for the ATX induction. Moreover, IFN-α/β can directly induce ATX in human PBMCs and monocytes through the IFNAR-mediated activation of JAK-STAT and PI3K-AKT pathways. A model of the mechanism for ATX expression regulation in response to TLR activation is presented as Fig 9. We propose that IFN-α/β produced by TLR activation functions as autocrine and paracrine factor to induce ATX expression in immune cells, leading to the enhanced LPA production in cellular microenvironment. Our data have provided intriguing and important bases for further elucidation of the mechanisms by which ATX-LPA axis is activated in inflammation responses.

## Supporting Information

S1 Fig The time- and dose-dependent induction of ATX and IFNα/β in THP-1 cells stimulated by TLR ligands. **(Figure A)** THP-1 cells were stimulated with LPS (0.1 μg/ml) for the indicated times. TNF-α, ATX, IFN-α and IFN-β mRNA expression were detected by RT-PCR. **(Figure B)** THP-1 cells were stimulated for with different concentration of LPS (0.01–1 μg/ml) as indicated. ATX mRNA expression was detected after LPS stimulation for 16h, while IFN-α/β mRNA expression were detected after LPS stimulation for 1h by RT-PCR. **(Figure C)** THP-1 cells were stimulated with CpG (1 μM) for the indicated times. ATX, IFN-α and IFN-β mRNA expression were detected by RT-PCR. **(Figure D)** THP-1 cells were stimulated for 6h with various amount of CpG (0.1–10 μM). ATX, IFN-α and IFN-β mRNA expression were detected by RT-PCR. **(Figure E)** THP-1 cells were stimulated with poly(I:C) (10 μg/ml) for the indicated times. ATX, IFN-α and IFN-β mRNA expression were detected by RT-PCR. **(Figure F)** THP-1 cells were stimulated for 6 h with various amount of poly(I:C) (1–50 μg/ml). ATX, IFN-α and IFN-β mRNA expression were detected by RT-PCR.
(TIFF)

S2 Fig The activation of JAK-STAT and PI3K-AKT pathways is essential for ATX induction in LPS-stimulated THP-1 cells. **(Figure A)** THP-1 cells were pretreated with LY294002 (10 μM) and pyridone 6 (P6; 10 μM) for 30 min, and then subjected to LPS treatment. After LPS treatment for 16 h, ATX mRNA levels were detected by qRT-PCR. **(Figure B)** THP-1 cells were transfected with AKT siRNA and non-specific siRNA (siNC) respectively. After siRNA transfection for 48 h, THP-1 cells were treated with LPS for 16 h. AKT protein was detected by Western blot, and ATX mRNA expression was analyzed by qRT-PCR. **(Figure C)** STAT1 and STAT3 siRNAs were transfected into THP-1 cells respectively, with non-specific siRNA (siNC) as the control. After siRNA transfection for 48 h, THP-1 cells were treated with LPS for 16 h. STAT1 and STAT3 were detected by Western blot, and ATX mRNA expression was analyzed by qRT-PCR. The ATX expression detected by qRT-PCR analyses was normalized to expression of GAPDH and presented relative to expression in untreated cells. All qRT-PCR data are expressed as mean values ± SD, n = 3. The p values derived from Student’s t test are (*) p < 0.05, (**) p < 0.01. A representative experiment out of three is shown.
(TIFF)

S3 Fig LPA production in response to IFN-α/β treatment and TLRs ligand stimulation in the presence of additional LPC. THP-1 cells were washed by PBS for three times and cultured with serum-free RPMI 1640, then stimulated by IFN-α (50 ng/ml) and IFN-β (10 ng/ml) respectively for 24 h **(Figure A)**, or by LPS (0.1 μg/ml) for 24 h, CpG ODN (1 μM) and poly(I:C) (10 μg/ml) respectively for 12 h **(Figure B)** in the presence of 18:1 LPC (100 μM) and 250μg/ml fatty-acid free BSA. After stimulation, 18:1 LPA levels in the supernatant of conditional medium were assayed by mass spectrometry. Data represent the mean and SD of triplicate determinations. The p values derived from Student’s t test are (*) p < 0.05, (**) p < 0.01.
(TIFF)

S4 Fig Western blot analysis of ATX in the conditional culture medium of THP-1 cells stimulated by TLR ligands. THP-1 cells were washed by PBS for three times and cultured with serum-free medium, then treated with TLR4 ligand LPS (0.1 μg/ml) for 24 h, TLR9 ligand CpG (1 μM) for 12 h, or TLR3 ligand poly(I:C) (10 μ/ml) for 12 h, respectively. The secreted ATX in the conditional culture medium was detected by Western blot.
(TIFF)

S5 Fig The effects of type I IFNs on ATX expression in different cell lines. **(Figure A)** IFNAR1 mRNA expression was detected by RT-PCR in HUVEC, Jurkat, HEK293, SW480, A549 and MCF-7 cells. **(Figure B)** HEK293, SW480, A549 and MCF-7 cells were treated with IFN-α (50 ng/ml) for 2 h or with IFN-β (10 ng/ml) for 4 h. ATX and MX2 mRNA expression levels were detected by RT-PCR.
(TIFF)

S6 Fig ATX is induced by TNF-α and IFN-γ together in THP-1 cells dependent the IFN-β autocrine loop. **(Figure A)** THP-1 cells were treated for 16h with TNF-α (50 ng/ml) and/or IFN-γ (50 ng/ml) as indicated. ATX mRNA levels were detected by qRT-PCR. **(Figure B)** THP-1 cells were preincubated with IFN-β specific neutralizing antibody (anti-IFN-β; 1μg/ml) or negative control antibody (rabbit IgG; 1 μg/ml) for 30 min, and then subjected to TNF-α and/or IFN-γ treatment as indicated. ATX mRNA levels were detected by qRT-PCR after 16 h treatment. **(Figure C)** IFNAR1 siRNA and non-specific siRNA (siNC) were transfected into THP-1 cells respectively. After siRNA transfection for 48 h, THP-1 cells were treated with TNF-α plus IFN-γ for 16 h. IFNAR1 were detected by Western blot, and ATX mRNA expression was analyzed by qRT-PCR. The ATX expression detected by qRT-PCR analyses was normalized to expression of GAPDH and presented relative to expression in untreated cells. **(Figure D)** THP-1 cells were treated with TNF-α (50 ng/ml) for 2 h, and then the expression of IFN-α and IFN-β mRNA was detected by RT-PCR. All qRT-PCR data are expressed as mean values ± SD, n = 3. The p values derived from Student’s t test are (*) p < 0.05, (**) p < 0.01. A representative experiment out of three is shown.
(TIFF)

S1 Table LPA levels in THP-1 cell culture medium with or without (control) IFN-α, IFN-β, LPS, CpG or poly(I:C) treatment. THP-1 cells were washed by PBS for three times and cultured with serum-free RPMI 1640, then stimulated by IFN-α (50 ng/ml), IFN-β (10 ng/ml) or LPS (0.1 μg/ml) for 24h and by CpG ODN (1 μM) or poly(I:C) (10 μg/ml) for 12h. The concentrations of 16:0, 18:0, and 18:1 LPA in the supernatant of conditional medium were assayed by mass spectrometry. Data represent the mean and SD of triplicate determinations. The p values derived from Student’s t test are (*) p < 0.05, (**) p < 0.01.
(DOCX)
