# Supplementary figures and images for: Type I Interferons Function as Autocrine and Paracrine Factors to Induce Autotaxin in Response to TLR Activation
Source: PLoS One. 2015 Aug 27;10(8):e0136629. doi: 10.1371/journal.pone.0136629 (PMC4552386; doi:10.1371/journal.pone.0136629)

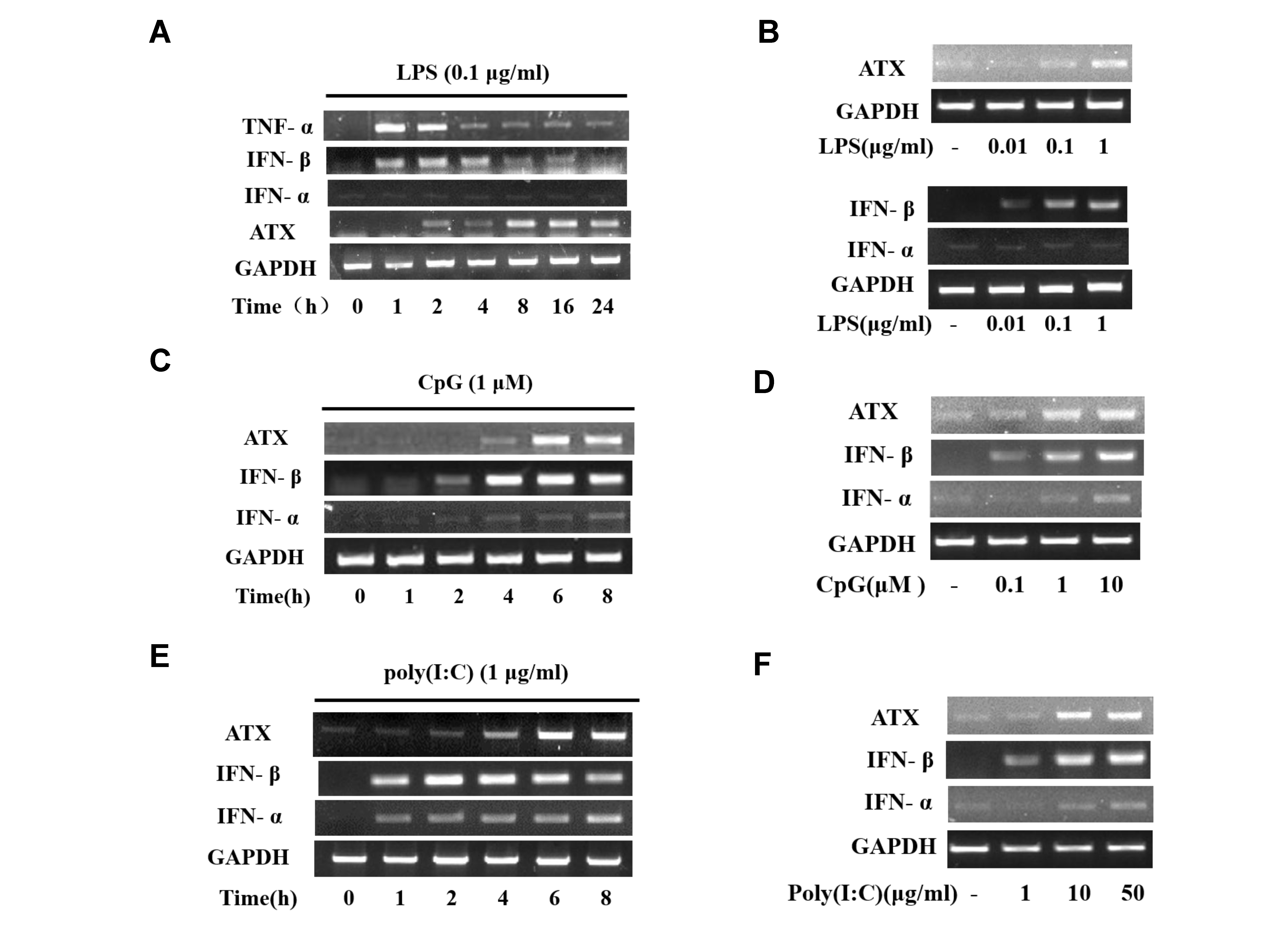

Supplement: S1 Fig — (Figure A) THP-1 cells were stimulated with LPS (0.1 μg/ml) for the indicated times. TNF-α, ATX, IFN-α and IFN-β mRNA expression were detected by RT-PCR. (Figure B) THP-1 cells were stimulated for with different concentration of LPS (0.01–1 μg/ml) as indicated. ATX mRNA expression was detected after LPS stimulation for 16h, while IFN-α/β mRNA expression were detected after LPS stimulation for 1h by RT-PCR. (Figure C) THP-1 cells were stimulated with CpG (1 μM) for the indicated times. ATX, IFN-α and IFN-β mRNA expression were detected by RT-PCR. (Figure D) THP-1 cells were stimulated for 6h with various amount of CpG (0.1–10 μM). ATX, IFN-α and IFN-β mRNA expression were detected by RT-PCR. (Figure E) THP-1 cells were stimulated with poly(I:C) (10 μg/ml) for the indicated times. ATX, IFN-α and IFN-β mRNA expression were detected by RT-PCR. (Figure F) THP-1 cells were stimulated for 6 h with various amount of poly(I:C) (1–50 μg/ml). ATX, IFN-α and IFN-β mRNA expression were detected by RT-PCR. (TIFF) [file pone.0136629.s001.tiff]

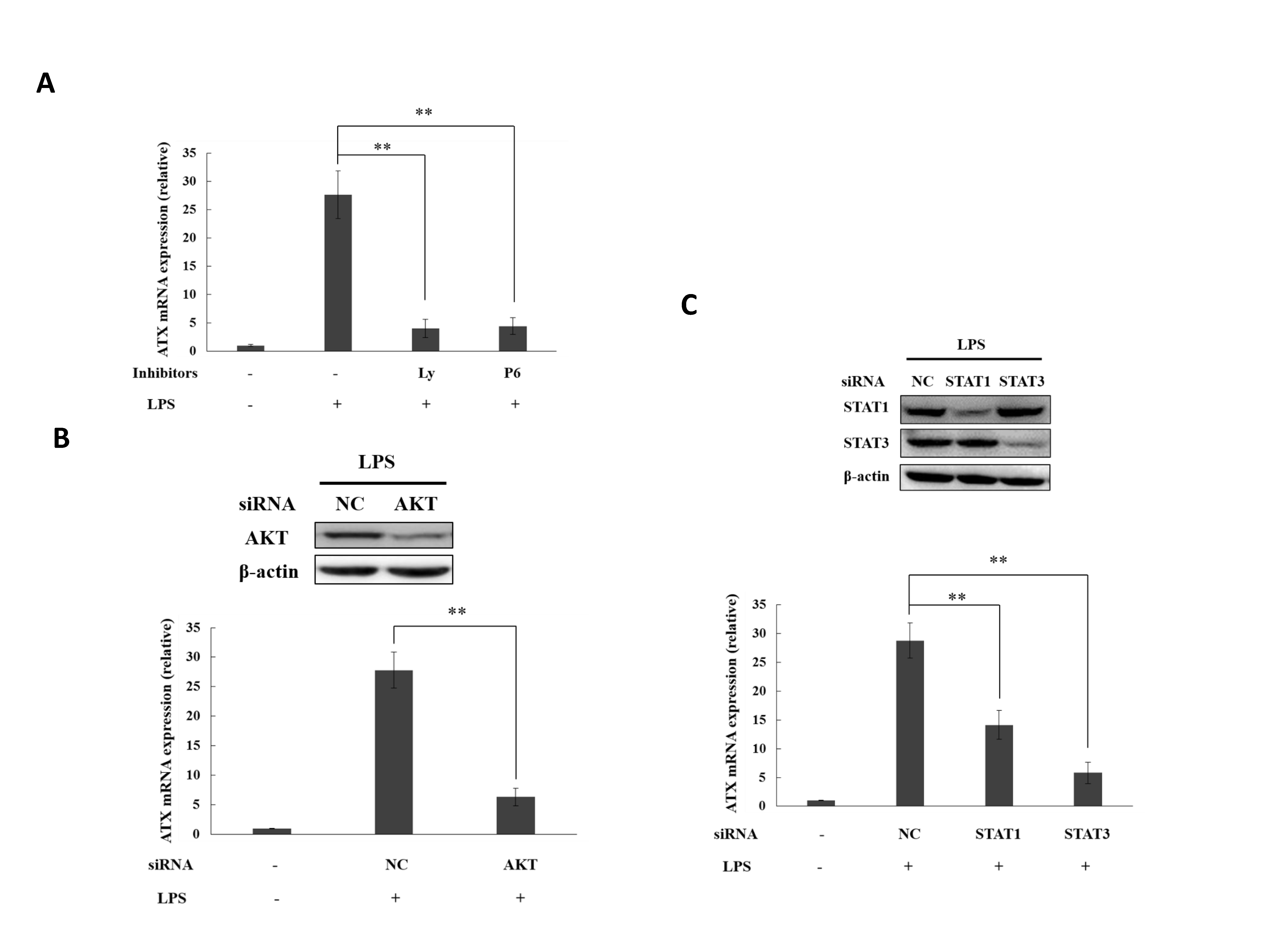

Supplement: S2 Fig — (Figure A) THP-1 cells were pretreated with LY294002 (10 μM) and pyridone 6 (P6; 10 μM) for 30 min, and then subjected to LPS treatment. After LPS treatment for 16 h, ATX mRNA levels were detected by qRT-PCR. (Figure B) THP-1 cells were transfected with AKT siRNA and non-specific siRNA (siNC) respectively. After siRNA transfection for 48 h, THP-1 cells were treated with LPS for 16 h. AKT protein was detected by Western blot, and ATX mRNA expression was analyzed by qRT-PCR. (Figure C) STAT1 and STAT3 siRNAs were transfected into THP-1 cells respectively, with non-specific siRNA (siNC) as the control. After siRNA transfection for 48 h, THP-1 cells were treated with LPS for 16 h. STAT1 and STAT3 were detected by Western blot, and ATX mRNA expression was analyzed by qRT-PCR. The ATX expression detected by qRT-PCR analyses was normalized to expression of GAPDH and presented relative to expression in untreated cells. All qRT-PCR data are expressed as mean values ± SD, n = 3. The p values derived from Student’s t test are (*) p < 0.05, (**) p < 0.01. A representative experiment out of three is shown. (TIFF) [file pone.0136629.s002.tiff]

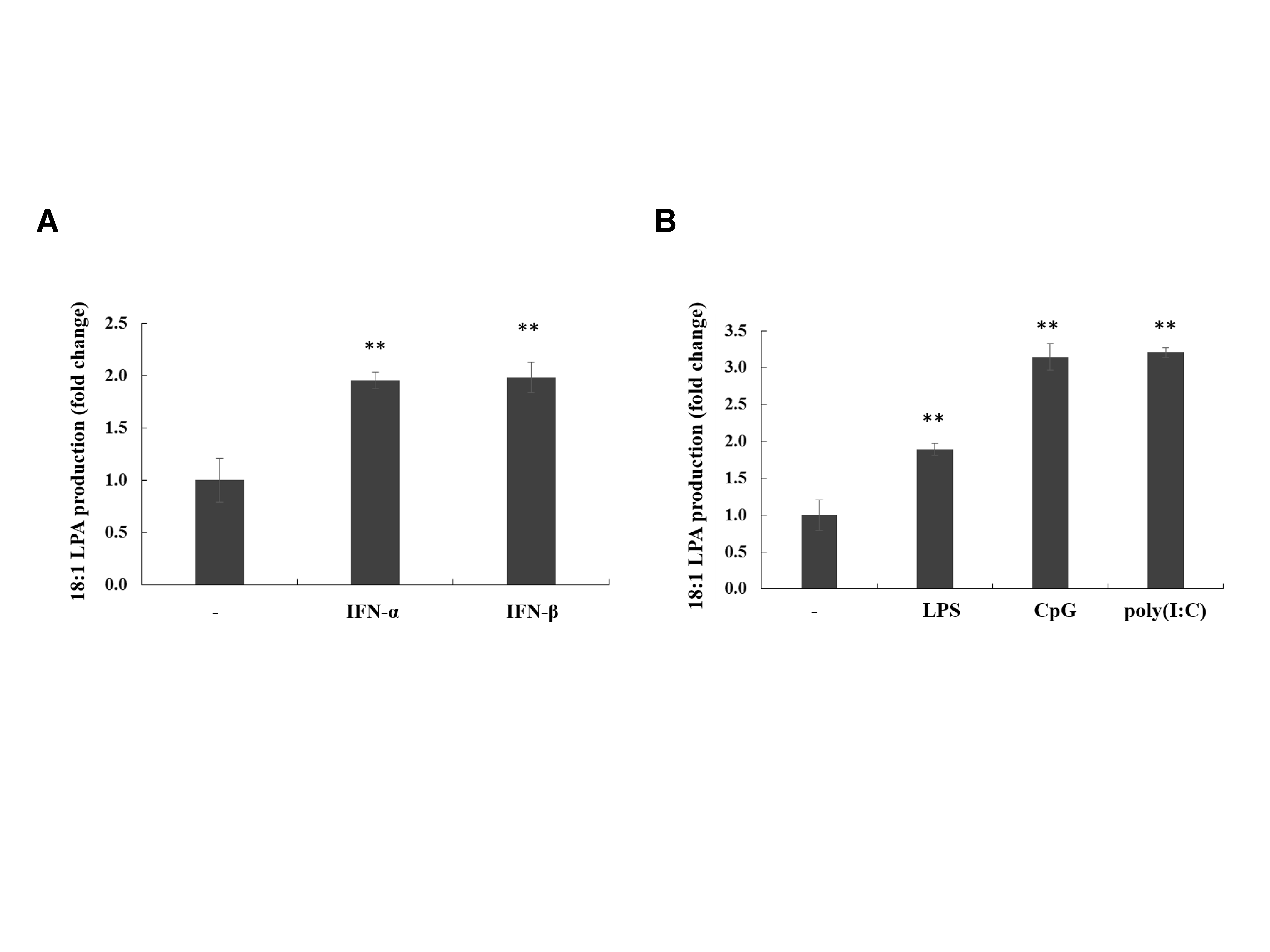

Supplement: S3 Fig — THP-1 cells were washed by PBS for three times and cultured with serum-free RPMI 1640, then stimulated by IFN-α (50 ng/ml) and IFN-β (10 ng/ml) respectively for 24 h (Figure A), or by LPS (0.1 μg/ml) for 24 h, CpG ODN (1 μM) and poly(I:C) (10 μg/ml) respectively for 12 h (Figure B) in the presence of 18:1 LPC (100 μM) and 250μg/ml fatty-acid free BSA. After stimulation, 18:1 LPA levels in the supernatant of conditional medium were assayed by mass spectrometry. Data represent the mean and SD of triplicate determinations. The p values derived from Student’s t test are (*) p < 0.05, (**) p < 0.01. (TIFF) [file pone.0136629.s003.tiff]

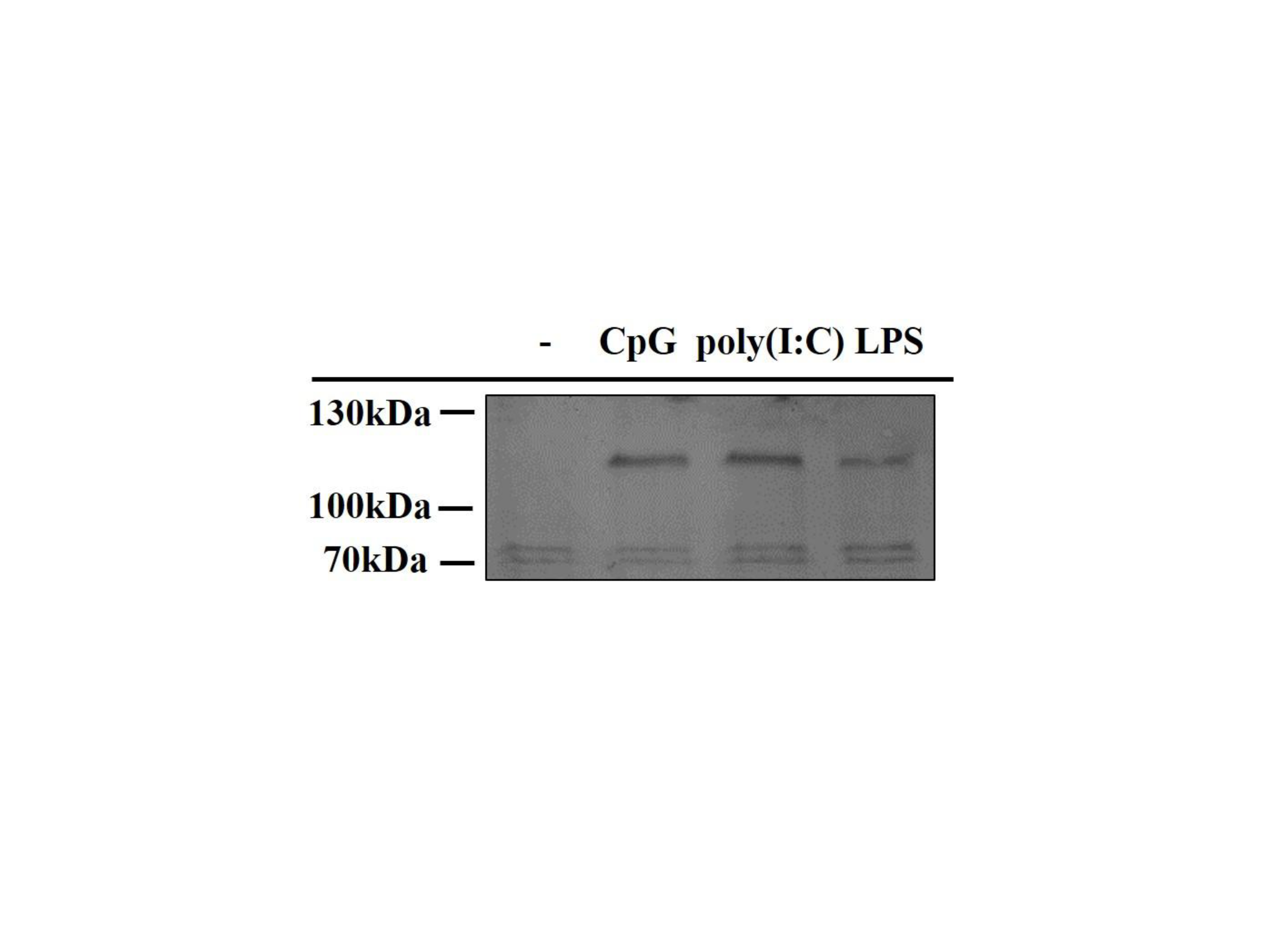

Supplement: S4 Fig — THP-1 cells were washed by PBS for three times and cultured with serum-free medium, then treated with TLR4 ligand LPS (0.1 μg/ml) for 24 h, TLR9 ligand CpG (1 μM) for 12 h, or TLR3 ligand poly(I:C) (10 μ/ml) for 12 h, respectively. The secreted ATX in the conditional culture medium was detected by Western blot. (TIFF) [file pone.0136629.s004.tiff]

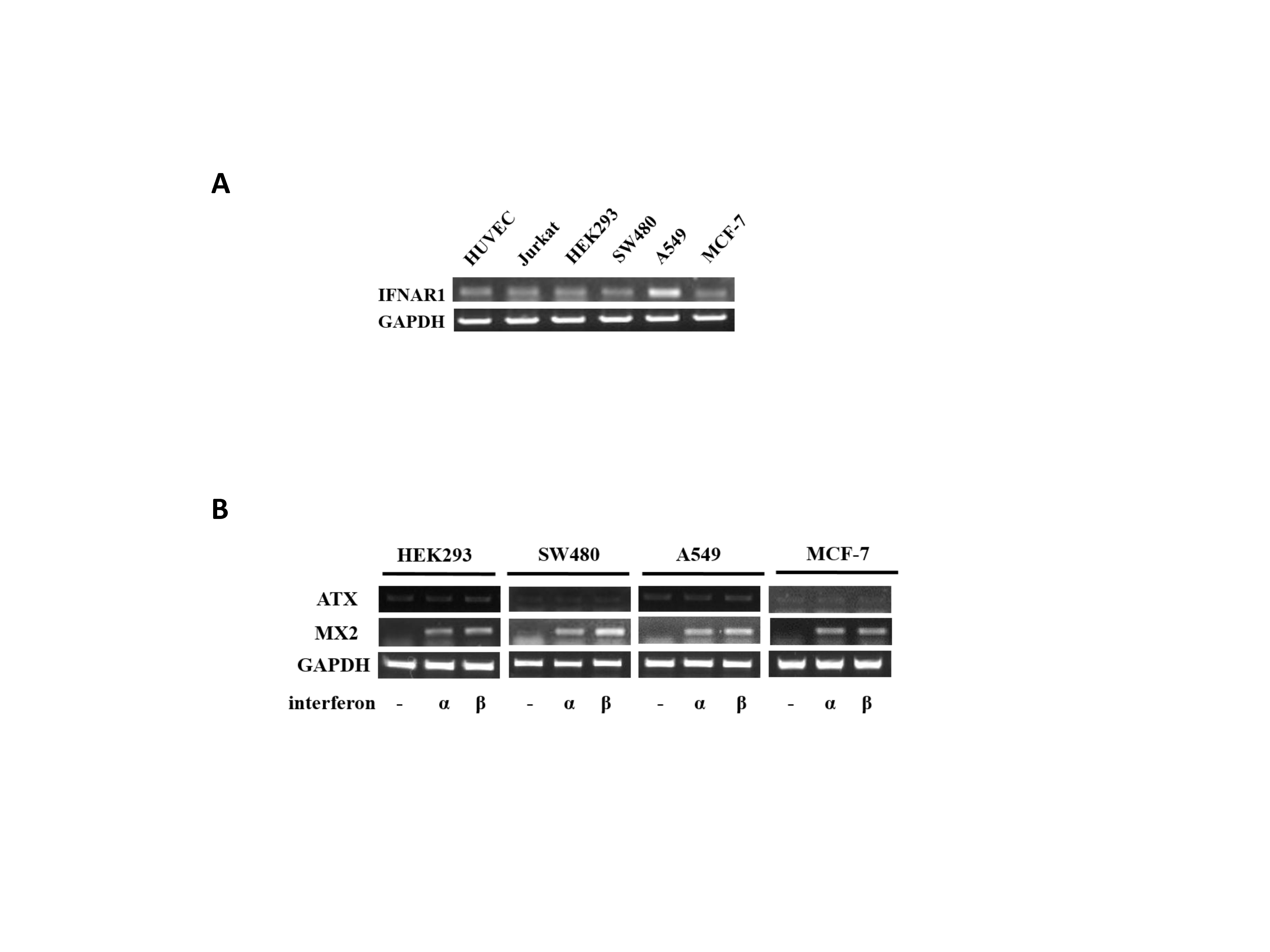

Supplement: S5 Fig — (Figure A) IFNAR1 mRNA expression was detected by RT-PCR in HUVEC, Jurkat, HEK293, SW480, A549 and MCF-7 cells. (Figure B) HEK293, SW480, A549 and MCF-7 cells were treated with IFN-α (50 ng/ml) for 2 h or with IFN-β (10 ng/ml) for 4 h. ATX and MX2 mRNA expression levels were detected by RT-PCR. (TIFF) [file pone.0136629.s005.tiff]

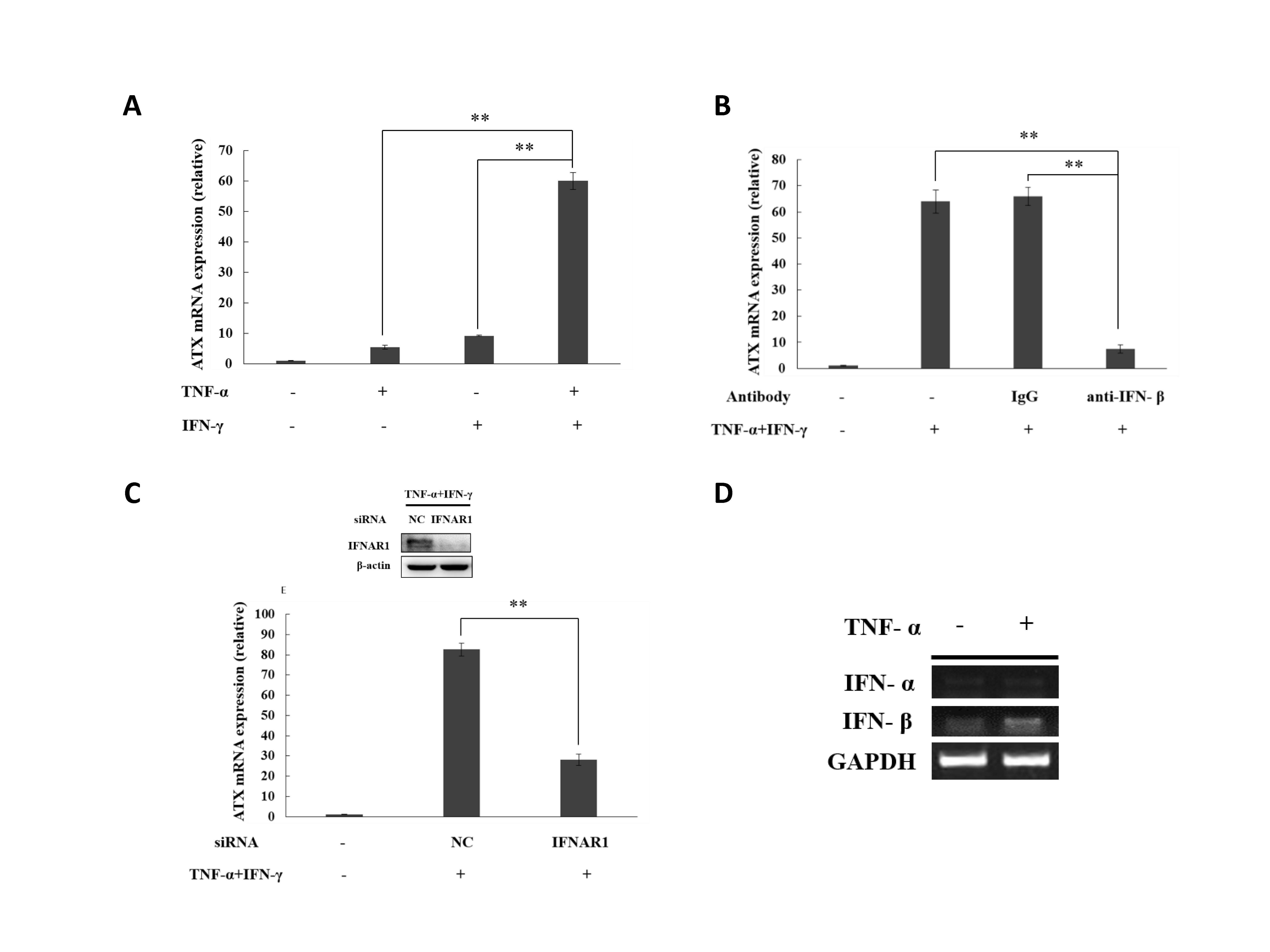

Supplement: S6 Fig — (Figure A) THP-1 cells were treated for 16h with TNF-α (50 ng/ml) and/or IFN-γ (50 ng/ml) as indicated. ATX mRNA levels were detected by qRT-PCR. (Figure B) THP-1 cells were preincubated with IFN-β specific neutralizing antibody (anti-IFN-β; 1μg/ml) or negative control antibody (rabbit IgG; 1 μg/ml) for 30 min, and then subjected to TNF-α and/or IFN-γ treatment as indicated. ATX mRNA levels were detected by qRT-PCR after 16 h treatment. (Figure C) IFNAR1 siRNA and non-specific siRNA (siNC) were transfected into THP-1 cells respectively. After siRNA transfection for 48 h, THP-1 cells were treated with TNF-α plus IFN-γ for 16 h. IFNAR1 were detected by Western blot, and ATX mRNA expression was analyzed by qRT-PCR. The ATX expression detected by qRT-PCR analyses was normalized to expression of GAPDH and presented relative to expression in untreated cells. (Figure D) THP-1 cells were treated with TNF-α (50 ng/ml) for 2 h, and then the expression of IFN-α and IFN-β mRNA was detected by RT-PCR. All qRT-PCR data are expressed as mean values ± SD, n = 3. The p values derived from Student’s t test are (*) p < 0.05, (**) p < 0.01. A representative experiment out of three is shown. (TIFF) [file pone.0136629.s006.tiff]
