# Supplementary material for: Type I Interferons Function as Autocrine and Paracrine Factors to Induce Autotaxin in Response to TLR Activation
Source: PLoS One. 2015 Aug 27;10(8):e0136629. doi: 10.1371/journal.pone.0136629 (PMC4552386; doi:10.1371/journal.pone.0136629)
Supplement: S1 Table — THP-1 cells were washed by PBS for three times and cultured with serum-free RPMI 1640, then stimulated by IFN-α (50 ng/ml), IFN-β (10 ng/ml) or LPS (0.1 μg/ml) for 24h and by CpG ODN (1 μM) or poly(I:C) (10 μg/ml) for 12h. The concentrations of 16:0, 18:0, and 18:1 LPA in the supernatant of conditional medium were assayed by mass spectrometry. Data represent the mean and SD of triplicate determinations. The p values derived from Student’s t test are (*) p < 0.05, (**) p < 0.01. (DOCX) [file pone.0136629.s007.docx]

**Table S1** LPA levels in THP-1 cell culture medium with or without (control) IFN-α, IFN-β, LPS, CpG or poly(I:C) treatment

|  | | **16:0 LPA(nM)**  **mean±SD** | **18:1 LPA(nM)**  **mean±SD** | **18:0 LPA(nM)**  **mean±SD** | **Tolal LPA(nM)**  **mean±SD** |
| --- | --- | --- | --- | --- | --- |
| **Control** | | 0.991±0.124 | 0.377±0.137 | 1.261±0.127 | 2.629±0.233 |
| **IFN-α** | | 1.269±0.108 | 0.866±0.189 | 1.317±0.044 | 3.571±0.208 |
| **IFN-β** | | 1.897±0.218 | 0.713±0.175 | 1.784±0.320 | 4.092±0.676 |
| **LPS** | | 1.431±0.008 | 0.643±0.078 | 1.492±0.105 | 3.642±0.189 |
| **CpG** | | 2.250±0.186 | 1.198±0.234 | 2.286±0.064 | 5.734±0.684 |
| **Poly(I:C)** | | 2.266±0.388 | 1.050±0.045 | 2.241±0.288 | 5.557±0.769 |
| ***P*** | **IFN-α vs control** | 0.0350 | 0.0220 | 0.2042 | 0.0254 |
|  | **IFN-β vs control** | 0.0175 | 0.0442 | 0.0782 | 0.0268 |
|  | **LPS vs control** | 0.0162 | 0.0348 | 0.0714 | 0.0189 |
|  | **CpG vs control** | 0.0029 | 0.0157 | 0.0019 | 0.0032 |
|  | **Poly(I:C) vs control** | 0.0057 | 0.0014 | 0.0059 | 0.0047 |

*p-value from the Student’s t test*
